# The primary transcriptome of the *Escherichia coli* O104:H4 pAA plasmid and novel insights into its virulence gene expression and regulation

**DOI:** 10.1038/srep35307

**Published:** 2016-10-17

**Authors:** Petya Berger, Michael Knödler, Konrad U. Förstner, Michael Berger, Christian Bertling, Cynthia M. Sharma, Jörg Vogel, Helge Karch, Ulrich Dobrindt, Alexander Mellmann

**Affiliations:** 1 Institute of Hygiene, University of Münster, Münster, Germany; 2 Core Unit Systems Medicine, University of Würzburg, Würzburg, Germany; 3 Institute for Molecular Infection Biology, University of Würzburg, Würzburg, Germany; 4 Research Center for Infectious Diseases, University of Würzburg, Würzburg, Germany

## Abstract

*Escherichia coli* O104:H4 (*E. coli* O104:H4), which caused a massive outbreak of acute gastroenteritis and hemolytic uremic syndrome in 2011, carries an aggregative adherence fimbriae I (AAF/I) encoding virulence plasmid, pAA. The importance of pAA in host-pathogen interaction and disease severity has been demonstrated, however, not much is known about its transcriptional organization and gene regulation. Here, we analyzed the pAA primary transcriptome using differential RNA sequencing, which allows for the high-throughput mapping of transcription start site (TSS) and non-coding RNA candidates. We identified 248 TSS candidates in the 74-kb pAA and only 21% of them could be assigned as TSS of annotated genes. We detected TSS for the majority of pAA-encoded virulence factors. Interestingly, we mapped TSS, which could allow for the transcriptional uncoupling of the AAF/I operon, and potentially regulatory antisense RNA candidates against the genes encoding dispersin and the serine protease SepA. Moreover, a computational search for transcription factor binding sites suggested for AggR-mediated activation of SepA expression, which was additionally experimentally validated. This work advances our understanding of the molecular basis of *E. coli* O104:H4 pathogenicity and provides a valuable resource for further characterization of pAA virulence gene regulation.

*Escherichia coli* O104:H4 (*E. coli* O104:H4) was identified as the causative agent for the 2011 outbreak centered in Northern Germany, in which nearly 4000 gastroenteritis cases were reported and more than 850 of them progressed to hemolytic uremic syndrome (HUS) leading to 54 deaths. This is the largest foodborne disease outbreak in German history and the highest incidence of *E. coli*-related HUS worldwide^1^,^2^. PCR-based genotypic analysis and whole genome sequencing revealed that with respect to virulence gene content *E. coli* O104:H4 is a hybrid of enterohemorrhagic (EHEC) and enteroaggregative *E. coli* (EAEC)^3^,^4^,^5^,^6^. The outbreak strain harbors a chromosomally integrated bacteriophage coding for the cardinal EHEC virulence factor Shiga toxin (Stx). In addition, *E. coli* O104:H4 carries the pAA virulence plasmid, which is characteristic for EAEC and encodes the aggregative adherence fimbriae I (AAF/I). The AAF/I confer a distinct “stacked- brick” aggregative adherence of EAEC and the 2011 outbreak strain to cultured human epithelial cells^6^,^7^. It was hypothesized that the tight intestinal adherence mediated by AAF/I facilitates systemic absorption of Stx and thus contributes to *E. coli* O104:H4 exceptional virulence^6^.

The role of pAA in *E. coli* O104:H4 pathogenicity has been addressed in several studies. On one hand, the fimbriae-encoding plasmid was found not to be essential for intestinal colonization of the outbreak strain in a rabbit model^8^. On the contrary, pAA loss sporadically observed during the course of the disease was correlated with a significantly reduced probability of HUS development in patients, which speaks for an attenuated virulence of the pAA-negative strain^9^. Furthermore, it was shown that the presence of pAA in *E. coli* O104:H4 promotes the translocation of Stx2 across an epithelial cell monolayer and enhances intestinal inflammation^10^. These observations demonstrate rather a central role of pAA in host-pathogen interaction and disease severity.

Besides the *aggDCBA* cluster encoding AAF/I, the 74-kb *E. coli* O104:H4 pAA plasmid harbors several other EAEC-specific virulence loci, e.g., *aap* coding for the dispersin surface protein mediating antiaggregation, the *aatPABCD* operon encoding the Aat transport system responsible for dispersin secretion, *sepA* coding for a homologue of the *Shigella flexneri* serine protease SepA, and a gene encoding the major EAEC virulence regulator AggR^3^,^4^,^5^,^6^. Dispersin was proposed to function in EAEC adhesion and intestinal colonization by allowing for proper fimbrial extension from the bacterial surface^11^,^12^. SepA mutants were associated with decreased mucosal inflammation in *Shigella* infection^13^ and with a significantly reduced IL-8 secretion from *E: coli* O104:H4 infected colonic epithelial monolayer^10^. AggR is an AraC-type transcriptional regulator which was found to positively regulate the expression of AAF/I, dispersin, the Aat secretion system and other pAA- as well as chromosomally encoded loci in EAEC^11^,^14^,^15^,^16^. AggR expression was described to be autoregulated, and activated and repressed by the nucleoid-associated proteins FIS and H-NS, respectively^17^.

Here, we analyzed the transcriptome profile of the pAA plasmid of the *E. coli* O104:H4 clinical isolate LB226692^3^,^6^ using differential RNA-sequencing (dRNA-seq), a terminator exonuclease (TEX)-based RNA-seq approach that allows for the discrimination of primary (5′-PPP) and processed (5′-P) transcripts^18^. While 5′-PPP ends are generated by the transcription process itself, 5′-P RNA ends result from the processing of primary transcripts by either RppH- and/or RNase-dependent mechanisms *in vivo*^19^,^20^. Here, we used the term processing site (PS) to define the coordinates of 5′-P RNA ends generated via processing. We catalogued pAA-associated transcription start site (TSS) and PS candidates on a plasmid-wide scale and performed a detailed analysis of the primary transcriptome focusing on known pAA virulence genes. In addition, we performed a computational-based screen for binding sites of the virulence regulator AggR in pAA. The predicted binding sites in close spatial proximity to the identified here *sepA* TSS suggested that *sepA* is part of the AggR regulon. We tested this hypothesis in a heterologous system and thus provided experimental evidence that AggR is indeed a positive regulator of *sepA*.

## Results

### dRNA-seq and mapping of transcription start sites and processing sites

**Figure 1:**
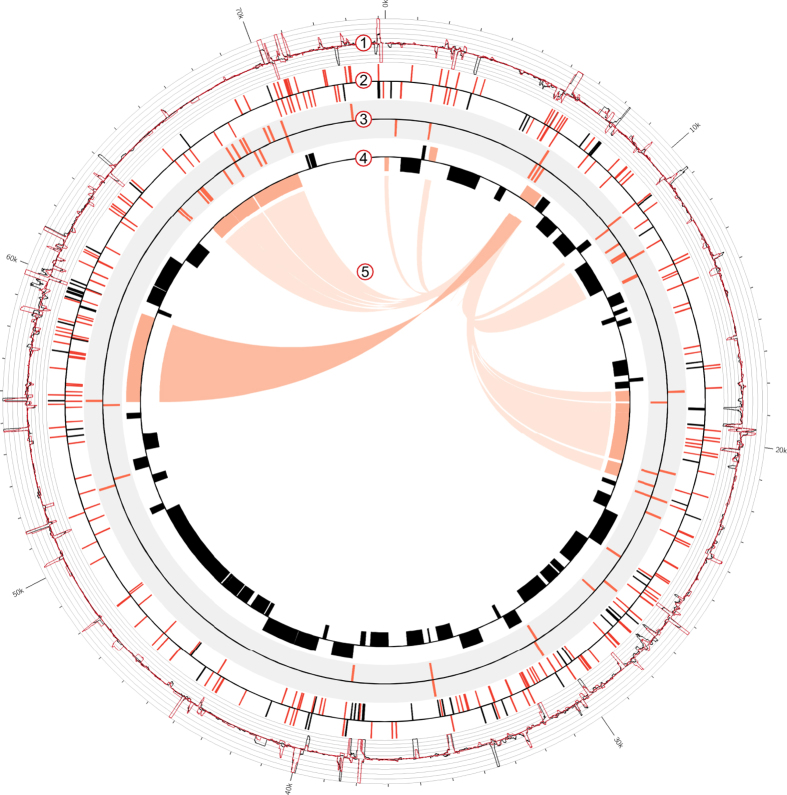
The transcriptome profile and the AggR regulon of the *E. coli* O104:H4 pAA plasmid. Track 1: dRNA-seq profile of the pAA plasmid. The graphs represent the normalized number of pAA reads mapped per nucleotide in TEX+ (red) and TEX- (black) libraries (y-axis = abundance relative score, max. 100). Track 2. TSS (red) and PS (black) candidates mapped by dRNA-seq. Track 3. Computationally predicted AggR (red) binding sites. Track 4. Annotated ORFs in pAA (NC_018666.1). Virulence associated genes are colored in orange. Track 5. AggR regulon. The AggR regulon is based on Morin *et al.*^16^ (light orange) and our study (dark orange). The image was generated using Circos^52^.

We employed dRNA-seq to map TSS and PS candidates in the *E. coli* O104:H4 isolate LB226692 during exponential growth phase (Supplementary Fig. S1). To discriminate between 5′-PPP and 5′-P transcripts two differential cDNA libraries were constructed. The control cDNA library (TEX-) was synthesized from untreated total RNA containing both 5′-PPP and 5′-P RNAs, whereas the library enriched for primary transcripts (TEX+) was generated from TEX-treated and thus 5′-P depleted RNA. In total, 54,408 and 109,883 reads were mapped on the pAA plasmid in TEX+ and TEX- library, which correspond to coverage of 59x and 118x, respectively (Supplementary Fig. S2). The pAA dRNA-seq profile is depicted in Fig. 1, Track 1. The characteristic redistribution of cDNA reads after the TEX treatment allowed for the identification of TSS and PS candidates. The 5′ termini, which are present in the TEX- sample, but absent or relatively reduced after the TEX treatment reveal the coordinates of 5′-P ends susceptible to the TEX treatment, i.e. PS^18^,^21^. The 5′ ends that appear relatively enriched after the TEX treatment are characteristic for primary transcripts and reveal TSS^18^.

We mapped 248 TSS and 79 PS candidates in the 74-kb pAA plasmid of *E. coli* O104:H4 (Fig. 1, Track 2 and Supplementary Dataset S1 and S2; for mapping criteria see Supplementary Methods). The TSS and PS candidates were subjected to experimental confirmation and comparison with literature data. A specific PCR product was obtained for 26 of the TSS and 10 of the PS selected for verification in a biological replicate by 5′ RACE (rapid amplification of cDNA ends), an alternative approach for the characterization of 5′ RNA ends (Supplementary Table S1). For 24/26 (92%) and 7/10 (70%) of the TSS and PS respectively, both 5′ end coordinates and phosphorylation status determined by 5′ RACE matched the dRNA-seq results. Three of the PS identified by dRNA-seq appeared as TSS in our 5′ RACE analysis (Supplementary Table S1; marked in red). Comparison with previously published data revealed that four of the TSS mapped by dRNA-seq (Supplementary Dataset S1, marked in bold) were in agreement with already described 5′-PPP ends, while two PS (Supplementary Dataset S2, marked in bold) were previously described as TSS in *E. coli* plasmids with homology to pAA^22^,^23^,^24^,^25^. The list of TSS was additionally subjected to computational validation. Stable RNA secondary structure in the vicinity of 5′-P ends can inhibit TEX activity and may therefore result in false positive TSS candidates^18^. However, RNA structure prediction analysis of the first 50 and 100 nucleotides of the putative pAA primary transcripts revealed a low probability for the occurrence of base pairings immediately downstream of the mapped TSS (Supplementary Fig. S3, see Supplementary Methods).

### A comprehensive analysis of the pAA primary transcriptome

**Figure 2:**
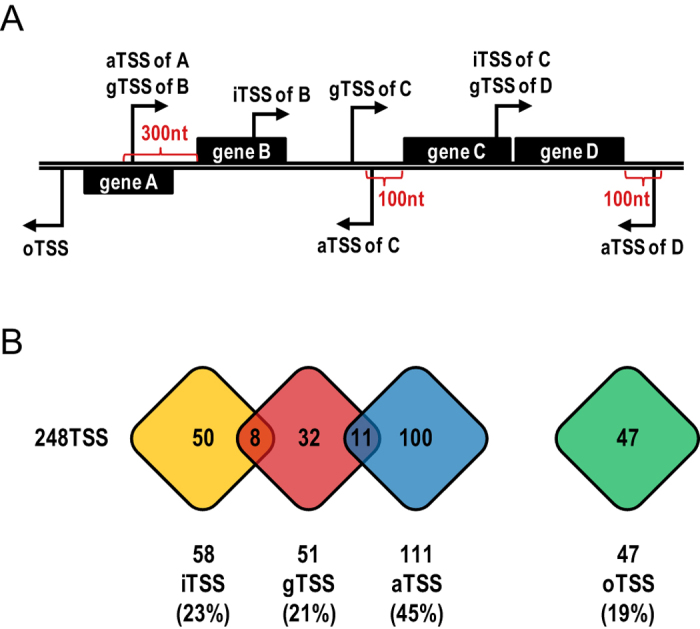
TSS categories. (**A**) Schematic diagram of assigned TSS categories. TSS candidates were classified into four categories based on their location in the pAA plasmid with respect to annotated ORFs: gene TSS (gTSS), internal TSS (iTSS), antisense TSS (aTSS) and orphan TSS (oTSS). (**B**) Overlap among TSS categories. The number of TSS candidates belonging to each category is presented. Several TSS could be assigned to two categories, i.e. gTSS and iTSS or gTSS and aTSS.

The obtained list of TSS candidates mapped by dRNA-seq (Supplementary Dataset S1) enabled us to perform a comprehensive analysis of the *E. coli* O104:H4 pAA primary transcriptome. Notably, TSS covering the whole plasmid were detected (Fig. 1, Track 2, red), indicating active sense and anti-sense transcription in regions coding for genes with known and unknown function, as well as in intergenic regions. Initiating nucleotide was in 77% (190/248 TSS) of the cases a purine, with adenine and guanine being equally represented. The TSS were classified into four categories based on their location with respect to annotated open reading frames (ORFs, Fig. 2A).

Fifty-one TSS candidates (21% of pAA-associated TSS; Fig. 2 and Supplementary Dataset S1) that mapped within the 300 nt upstream region of annotated ORFs were designated as gene TSS (gTSS) and most likely represent TSS participating in pAA mRNA synthesis. The average 5′UTR length was found to be 124 nt, 15 nt longer than the one described for the *E. coli* K-12 MG1655 chromosome^26^. Using the predicted operon organization available in the Database of prokaryotic operons DOOR^27^, which was additionally corrected based on experimental data available in the literature (see Supplementary Methods), we consider that 44 genes are clustered into 16 operons and 37 genes are transcribed monocistronically in pAA of *E. coli* O104:H4. At least one gTSS was detected for 5 operons and 22 monocistronic genes in our analysis. Interestingly, 10 gTSS were mapped within 7 operons, indicating potential transcriptional uncoupling of these gene clusters (Supplementary Table S2, marked in red).

TSS, which were mapped within annotated ORFs, were rated as internal TSS (iTSS; Fig. 2A). We detected 58 iTSS candidates which represented 23% of all mapped pAA TSS (Fig. 2B and Supplementary Dataset S1). Thirty-two pAA genes (40%) were associated with at least one internal TSS. Only eight of the iTSS (14%) were additionally annotated as gTSS.

The 111 TSS candidates found antisense to annotated ORFs or within the 100 nt upstream or downstream region of ORFs were rated as antisense TSS (aTSS; Fig. 2 and Supplementary Dataset S1). This was the most abundant TSS category, comprising 45% of all mapped TSS, suggesting extensive cis-encoded antisense RNA (asRNA) synthesis in the pAA plasmid. aTSS were mapped against 51 pAA genes (63%), with 32 of them found to be associated with multiple antisense transcripts. The 3′ ends of four selected asRNA candidates were determined by 3′ RACE and the length of the antisense transcripts was found to be in between 60 and 150 nt (Supplementary Table S3).

The remaining 47 TSS (19% of all TSS) were found in intergenic regions and could not be assigned to any of the above described categories. These TSS candidates were rated as orphan TSS (oTSS, Fig. 2 and Supplementary Dataset S1). Three oTSS were selected for 3′ RACE and the corresponding ncRNA candidates were determined to be in between 70 and 260 nt long (Supplementary Table S3).

### Promoter analysis and search for transcriptional regulator binding sites

**Figure 3:**
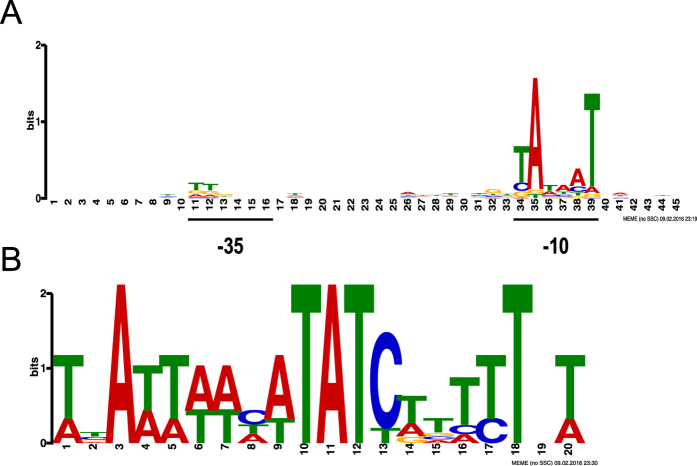
Computational motif analysis. (**A**) Promoter motif detected in the pAA plasmid of exponentially growing *E. coli* O104:H4. The sequence logo represents the promoter motif discovered by MEME within the 50 bp upstream region of the 248 TSS mapped by dRNA-seq. (**B**) AggR consensus binding motif. The sequence logo was generated by aligning the known experimentally determined Rns and AggR binding sequences^17^,^32^,^33^ and used for the prediction of AggR binding sites in pAA using FIMO.

A MEME^28^ (Multiple Em for Motif Elicitation) search for consensus promoter elements within the 50-bp region upstream of the TSS candidates mapped by dRNA-seq revealed a sequence (tAtaaT; Fig. 3A), resembling the −10 hexamer (TATAAT) recognized by sigma70 (σ^70^), the major sigma factor in *E. coli* in exponential growth phase^29^. For 225/248 (91%) of the TSS candidates the −10 hexamer was found centered 8–11 bp upstream of the mapped TSS. The −35 hexamer (TTGACA), however, was found to be overall poorly conserved in the pAA promoter regions (Fig. 3A), which is in agreement to what has been reported for *E. coli* K-12 chromosomal sigma 70-dependent promoters^30^. Both −10 and −35 hexamers with at least 3/6 nt match to the consensus sequences were manually identified in the putative promoter regions of all known virulence-associated genes and genes involved in plasmid replication, maintenance and segregation with a gTSS mapped in our analysis (Supplementary Dataset S1, sequences underlined).

In addition, we performed a computational-based search for putative binding sites of the pAA transcriptional regulator AggR. AggR was shown to substitute for Rns, another AraC-type activator which is involved in virulence gene regulation in enterotoxigenic *E. coli*^31^. Here, an AggR consensus binding sequence (Fig. 3B) was defined by aligning the experimentally determined five Rns and two AggR binding sites^17^,^32^,^33^. Next, the consensus sequence was used to search for potential AggR binding sites in pAA of *E. coli* O104:H4 using FIMO^34^ (Find Individual Motif Occurrence). 57 binding sites with a p-value < 0.0001 were predicted (Fig. 1, Track 3, red and Supplementary Table S4). At least one putative AggR binding site could be assigned to all *E. coli* O104:H4 homologues of the pAA genes described to be part of the AggR regulon in EAEC^14^,^16^,^17^, demonstrating the accuracy of our computational prediction. Moreover, the predicted AggR binding sequences were found significantly more often associated with known virulence-associated gene than with non-virulence genes (p = 9.25e-07, Fisher exact test, n = 81 genes, see Supplementary Methods), revealing a clear correlation of predicted AggR binding site occurrence and genes function.

### Expression and regulation of pAA virulence-associated genes

**Figure 4:**
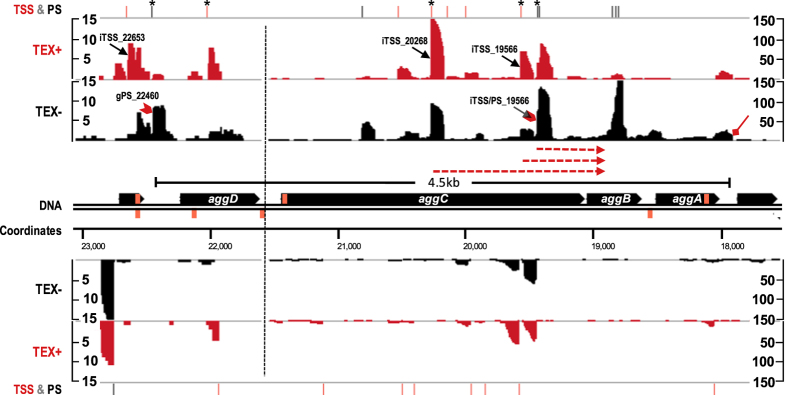
The transcriptome profile of AAF/I operon. cDNA reads from TEX+ (red) and TEX- (black) libraries mapped against the *aggDCBA* operon were visualized with the Integrated Genome Browser. Due to differential abundance of AAF/I-associated cDNA reads the image is split in two parts (dashed vertical line) and the y axis representing the abundance relative score for each part is given. Annotated TSS (red) and PS (black) are shown above the dRNA-seq graphs and the ones subjected to 5′ RACE verification in a biological replicate are marked with an asterisk. TSS and PS discussed in the main text are indicated in the dRNA-seq graphs with black arrows and red arrow heads, respectively. The transcripts of three *aggC* iTSS, which were detected to reach the downstream *aggB* in 5′-RACE analysis are depicted by red dashed arrows below the dRNA-seq graphs. The 3′ end of the operon determined by 3′ RACE is indicated by red diamond arrow. The calculated length of the *aggDCBA* mRNA (black line segment) is given. The predicted AggR binding sites are represented on the DNA strand as orange boxes.

The dRNA-seq profile in combination with the predicted AggR binding sites provided a detailed snapshot of pAA virulence gene expression and regulation in *E. coli* O104:H4. Only a gPS (PS, mapped within the 300 nt upstream region) at position 22460 (gPS_22460) was detected for the AAF/I operon (*aggDCBA*) in our analysis (Fig. 4, Supplementary Dataset S2 and Table S1). An iTSS at position 22653 (iTSS_22653) was mapped 405 nt upstream *aggD*, but 5′ RACE analysis failed to detect it as a TSS of the fimbrial operon. Interestingly, transcripts initiating from two of the multiple *aggC*-associated iTSS mapped by dRNA-seq (iTSS_19566 and iTSS_20268) and one mapped in 5′ RACE analysis (iTSS/PS_19439, mapped as a PS by dRNA-seq) were detected to reach the downstream *aggB* gene (Supplementary Table S1). Thus, these TSS candidates could be additionally regarded as operon-internal gTSS. In addition, eight aTSS were mapped against the AAF/I operon. The 3′ RNA end of the operon was determined by 3′ RACE and the full length of the *aggDCBA* polycistronic mRNA was found to be ~4.5 kb. Our computational analysis detected two potential AggR binding sites upstream the AAF/I cluster (Supplementary Table S4).

AggR was found to be expressed from gTSS_6649 and gTSS_6739 mapped 216 and 126 nt, respectively, upstream of its ORF (Supplementary Fig. S4 and Dataset S1). In addition, an abundant TSS (oTSS_6357) was detected 508 nt upstream of the *aggR* start codon. 3′ RACE was performed in the *aggR* upstream region in order to check if this oTSS candidate is involved in ncRNA synthesis or if it participates in *aggR* expression as a distant gTSS. Indeed, the 3′ ends of transcripts originating from this upstream region were not found to reach the *aggR* ORF (Supplementary Table S3). Four AggR binding sites were detected upstream and within *aggR* (Supplementary Table S4), two of which were already shown to be functional in the EAEC strain 042^17^.

Recently, it was shown that pAA of EAEC strain 042 carries a gene coding for the AggR-activated regulator Aar^35^. In this study, only a processed mRNA end (gPS_195) 31 nt upstream of the *E. coli* O104:H4 homologue was detected by both dRNA-seq and 5′ RACE (Supplementary Dataset S2 and Table S1). An AggR binding site was predicted 215 nt upstream of the *aar* 5′-P mRNA end (Supplementary Table S4).

Dispersin was found to be transcribed from two adjacent gTSS (gTSS_1987 and gTSS_1994, Supplementary Fig. S5, Dataset S1 and Table S1). An AggR binding site upstream of these promoters was predicted. Interestingly, the most abundant asRNA candidate detected in our analysis (aTSS_2433) was mapped against *aap*. The asRNA was found to be 90 nt long (Supplementary Table S3) and represented by approx. 100 times more reads than the sense RNA in our dataset.

Besides having an upstream TSS (gTSS_70088), the gene cluster *aatPABCD* encoding the Aat transport system was found associated with two operon-internal gTSS, gTSS_67507 upstream *aatB* and gTSS_66404 upstream *aatD* (Supplementary Fig. S6, Dataset S1 and Table S1). Moreover, 16 AggR putative binding sites were predicted upstream or within the operon (Supplementary Table S4), which is the highest concentration of putative binding sites in our analysis and reflects the relatively rich AT sequence of the *aatPABCD*.

The gene encoding the serine protease SepA was found to be the pAA gene associated with the highest number of aTSS candidates (n = 15, Supplementary Fig. S7 and Dataset S1). Interestingly, two AggR binding sites were computationally predicted in proximity to the mapped *sepA* gTSS_55560 (Supplementary Table S4), suggesting an AggR-dependent regulation of SepA expression which was not described so far.

### Regulation of SepA expression

**Figure 5:**
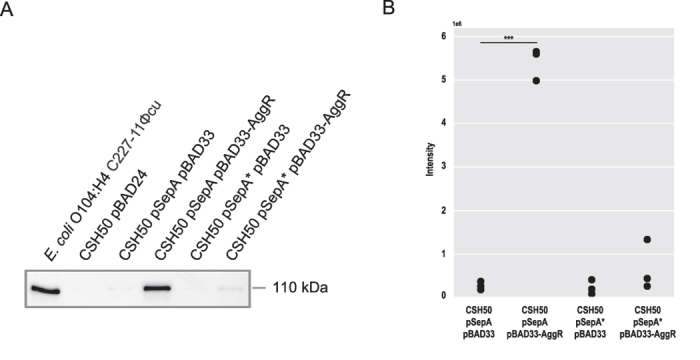
AggR-dependent SepA regulation. (**A**) Semi-quantitative Western blot analysis of AggR-dependent *sepA* activation. Culture supernatants of the *E. coli* O104:H4 strain C227-11Φcu (+control) and *E. coli* K-12 CSH50 containing the plasmids pBAD24 (-control), pSepA pBAD33, pSepA pBAD33-AggR, pSepA* pBAD33 and pSepA* pBAD33-AggR were subjected to semi-quantitative Western blot analysis with anti-SepA antibody. The full-length immunoblot is presented in Supplementary Fig. S8A **(B**). Quantification of AggR-dependent *sepA* activation in biological replicates. Signal intensities of bands of interest were quantified by Image Lab Software and dot plot diagrams summarizing the results from three biological replicates (Supplementary Fig. 8B) were generated using Python (***p < 0.001).

To experimentally test the hypothesis that AggR regulates SepA expression we cloned *sepA* under the control of its native promoter and *aggR* under an arabinose inducible promoter in plasmids pSepA and pBAD33-AggR, respectively (see Supplementary Methods). Both computationally predicted AggR binding sites were present in the cloned *sepA* region. We then measured SepA production when expressed from the plasmid in *E. coli* K-12 alone (CSH50 pSepA pBAD33) or in combination with *aggR* (CSH50 pSepA pBAD33-AggR) by semi-quantitative Western blot analysis (Fig. 5A). The SepA amount was found on average 20-fold higher (p = 2.03e-05, two-tailed Student t-test, n = 3 biological replicates) when AggR was co-expressed in *E. coli* K-12 (Fig. 5B). In addition, we measured the expression of SepA from a plasmid with a truncated upstream region (pSepA*), which did not include the high-scoring AggR binding site predicted upstream of the *sepA* gTSS (see Supplementary Methods). As expected, only a relatively small and not significant AggR-dependent activation of sepA was detected in the absence of the upstream binding site (on average 3-fold higher expression in the presence of AggR, p = 0.26, two-tailed Student t-test, n = 3 biological replicates, Fig. 5B). These results indicate that AggR is a transcriptional activator of *sepA* (Fig. 1, Track 5, dark orange) and the predicted binding site upstream of *sepA* TSS is an important determinant of the AggR-dependent regulation.

## Discussion

The pAA plasmid plays a central role in *E. coli* O104:H4 pathogenicity^9^,^10^. Here, we investigated the pAA primary transcriptome with focus on virulence-associated genes using dRNA-seq, a powerful method for mapping of TSS and ncRNAs in bacteria^36^. Moreover, we expanded the knowledge on the impact of the transcriptional activator AggR on pAA virulence gene expression.

Here, we used dRNA seq to map both TSS and PS candidates in the pAA virulence plasmid. The vast majority of tested candidates could be confirmed by 5′ RACE, an alternative method to analyze the 5′ phosphorylation status of RNAs. In three cases, however, we observed that the same 5′ mRNA end was detected by dRNA-seq to be in a 5′-P and by 5′-RACE in a 5′-PPP form (Supplementary Table S1; marked in red). A possible explanation for the discrepancies between the two methods may be that these 5′ ends exist in significant fractions both in a 5′-PPP and 5′-P form *in vivo*. Indeed, such a dual state of 5′mRNA ends has been already described for several targets of the pyrophosphohydrolase RppH^37^, which converts 5′-PPP to 5′-P and thus initiates the 5′ end-dependent mRNA decay in bacteria^38^.

The dRNA-seq analysis revealed a high complexity of the pAA primary transcriptome in *E. coli* O104:H4. The number of mapped pAA TSS candidates (248 TSS) was much higher than what may be expected for a 74-kb plasmid carrying 81 annotated ORFs. However, only 21% of the pAA TSS were assigned as TSS of annotated genes, which is similar to what is described for the chromosomal primary transcriptome of exponentially growing *E. coli* K-12 strain^26^. This percentage might be slightly higher, since promoters mapped beyond the 300 nt upstream region of annotated ORFs, which was used here as a cut-off distance for gTSS annotation, were described to drive mRNA synthesis in *E. coli* as exceptions rather than the rule^39^. Indeed, transcripts of several distant pAA TSS candidates were detected to reach the corresponding downstream genes by 5′ RACE (Supplementary Table S1).

The dRNA-seq analysis identified gTSS for the majority of virulence-associated genes and operons suggesting that they are expressed at least on the mRNA level in *E. coli* O104:H4 under the experimental conditions tested. Only a 5′-P mRNA end for the *aar* gene (gPS) was detected by both dRNA-seq and 5′ RACE (Supplementary Dataset S1 and Table S1). However, the mapped *aar* gPS is located directly downstream of the promoter region shown to drive its expression in the EAEC strain 042^35^. Since these regions are identical on the nucleotide level in both strains, one possibility is that under certain conditions the *aar* 5′-P end also exists as a primary transcript in pAA of *E. coli* O104:H4, but may not be detectable in exponentially growing cells due to high mRNA turnover rates.

The organization of genes in an operon guarantees for their coordinated gene expression^40^. However, internal promoters which are a common feature of *E. coli* operons^41^, increase transcriptional complexity by allowing distal gene expression independent of the primary operon-upstream promoter. Here, we detected the existence of operon-internal TSS candidates, e.g, within the gene clusters encoding AAF/I and the Aat transport system responsible for dispersin secretion (Supplementary Dataset S1 and Table S1). Intriguingly, the three AAF/I internal TSS were mapped within the *aggC* gene and thus could provide the possibility for the transcriptional uncoupling of the secreted AAF/I protein subunits AggA and AggB from the outer membrane usher protein AggC and periplasmic chaperone AggD.

There is emerging evidence for the important role of asRNA in virulence gene regulation^42^,^43^. Here, all known pAA-encoded virulence genes and operons except *aggR* were found to be associated with at least one asRNA candidate. Moreover, the most prominent examples in our analysis were found to be virulence gene-associated: the most abundant asRNA candidate was mapped against the dispersin gene and *sepA* was the pAA gene with the highest number of asTSS (Supplementary Dataset S1). The actual contribution of these asRNAs to virulence gene expression in *E. coli* O104:H4 is under current investigation.

We detected unusually high transcriptional activity in intergenic regions of the pAA plasmid in *E. coli* O104:H4 in comparison to other dRNA-seq datasets^18^,^26^,^44^. Disrupted ORFs resulting from insertion/deletion events in pAA could partially explain the high number of oTSS candidates. For example, the TSS of the *traJ* gene and the *finP* asRNA, which participate in the control of the F transfer region in plasmid R1^24^, were both mapped as oTSS in the pAA plasmid where this region is truncated^5^,^45^ (Supplementary Dataset S1). Nevertheless, it might be interesting to further investigate if oTSS-associated transcripts could play a role as regulatory ncRNAs in *E. coli* O104:H4 gene expression.

Homologs of all known virulence genes previously reported to be part of the AggR regulon in EAEC^14^,^16^ were found to be associated with at least one computationally predicted AggR binding site in the pAA plasmid of *E. coli* O104:H4 (Supplementary Table S4). In addition, the two AggR binding sites predicted in close proximity to the *sepA* TSS suggested AggR-dependent *sepA* regulation, which was not described so far. Indeed, the expression of *sepA* in *E. coli* K-12 from a plasmid carrying the gene and the predicted AggR binding sites was found approx. 20-fold stronger on the protein level in the presence of AggR (Fig. 5), indicating that AggR is an activator of *sepA*. Moreover, the exclusion of the predicted high-scoring upstream binding site resulted in a dramatic reduction in the AggR-dependent *sepA* activation (from 20- to 3-fold). The relatively small and not significant, but still measurable activation of *sepA* in the absence of the upstream binding site may be explained by a minor AggR-dependent activation mediated by an interaction with the second, lower-scoring site predicted downstream of the *sepA* TSS (Supplementary Table S4). Indeed, both AggR and Rns have already been shown to recognize regions upstream and downstream of TSS^17^,^31^,^33^. Taken together, our data provides strong evidence that the serine protease SepA is a member of the AggR regulon. All known virulence factors encoded on the pAA plasmid of *E. coli* O104:H4 are thus placed under the control of AggR (Fig. 1, Track 5), which may be functionally important to ensure their coordinated expression during infection.

In summary, we performed a comprehensive analysis of the pAA primary transcriptome and delivered novel insights into its virulence gene expression and regulation. Thus, this study advances our understanding of the molecular basis of *E. coli* O104:H4 pathogenicity, as well as of EAEC strains harboring homologous pAA plasmids, and provides a valuable resource for further characterization of pAA virulence gene regulation.

## Methods

### Bacterial strains

The transcriptome analyses was performed with the *E. coli* O104:H4 clinical isolate LB226692^3^,^6^. Heterologous expression of *sepA* and *aggR* was carried out in *E. coli* K-12 strain CSH50^46^. The *E. coli* O104:H4 strain C227-11Φcu (cured of Stx2a encoding phage)^47^ was used as a positive control in SepA Western blot analysis.

### Bacterial growth and RNA preparation for transcriptome analysis

An overnight culture of *E. coli* O104:H4 strain LB226692 was diluted 1:10,000 in LB medium (10 g/L tryptone, 5 g/L yeast extract, 5 g/L NaCl) and cells were grown at 37° C, 180 rpm for 3.5 h to an OD_600_ of 0.5–0.6 (Supplementary Fig. S1). 5 ml of cell suspension were added to 0.625 ml of pre-chilled stop solution (5% phenol/95% ethanol), vortexed and incubated for 5 min on ice. Cells were pelleted for 5 min at 5,000 g, flash frozen in a dry ice-ethanol bath and stored at −80° C. Pelleted cells were thawed on ice and lysed with 70 μL of cell lysis buffer (85 mg/mL lysozyme in TE buffer, pH 8). Total RNA was extracted using the Trizol reagent (Invitrogen) and the concentration and purity of the samples were determined using Nano Drop (Thermo Fisher Scientific). RNA integrity was monitored using the R6K ScreenTape system on the Agilent 2200 TapeStation. gDNA was removed by Turbo DNase (Ambion) in the presence of 1U/μL RiboLock RNase Inhibitor (Fermentas) for 1 h. Following organic extraction (1 × 25:24:1 v/v phenol/chloroform/isoamyalcohol, 1x chloroform), RNA was recovered by overnight precipitation with 3 volumes of 30:1 100% ethanol/3 M sodium acetate (pH 5.2). Real time PCR and RT-PCR with *gapA* specific primers were performed to control the efficiency of the gDNA removal and for presence of inhibitors, respectively. RNA was extracted from two biological replicates; one was used for dRNA-seq and the other one for 5′ and 3′ RACE analysis.

### dRNA-seq: depletion of processed RNAs, cDNA library construction and sequencing

The dRNA-seq experimental setup is described in details in Supplementary Methods and outlined in Supplementary Fig. S2. Depletion of processed RNAs and cDNA library construction were performed by vertis Biotechnology AG, Germany. Briefly, genomic DNA-free total RNA was depleted of processed RNAs with TEX (Epicentre) as described previously^18^. cDNA libraries for the Illumina sequencing platform were constructed as described previously^48^, without the RNA size-fractionation step prior to cDNA synthesis. Sequencing was performed on a HiSeq 2500 machine (Illumina) in a 100 bp single-end read mode at the Max Planc-Genome-centre Cologne, Germany.

### dRNA-seq data analysis: read mapping, data visualization and TSS and PS annotation

Briefly, rRNA and tRNA reads were filtered out after linker and poly A-tail removal. The resulting reads for the TEX+ and TEX- library, respectively, were mapped to the *E. coli* O104:H4 genome consisting of the chromosome and the plasmids pESBL, pG and pAA using READemption version 0.3.7^49^ and segemehl version 0.2.0^50^. For each library, graphs representing the number of mapped reads per nucleotide in pAA (NCBI accession number NC_018666.1) were calculated and visualized using the Integrated Genome Browser version 8.2^51^. To allow for comparison between the differential libraries, graphs were normalized to the total number of *E. coli* O104:H4 reads mapped per library. Annotation of TSS and PS was performed with the TSSPredator program (http://it.inf.uni-tuebingen.de/TSSpredator)^44^. For further details, please refer to Supplementary Methods.

### Sequence analysis: promoter mapping and computational prediction of AggR binding sites in pAA

Briefly, the 50-bp upstream regions of the 248 TSS mapped by dRNA-seq were screened with MEME^28^ for conserved promoter motifs with fixed width or in the range of 6 to 45 nt. The search with a fixed width of 45 nt resulted in a motif (Fig. 3A), which could be detected in all 248 analyzed sequences. The 20-nt long AggR consensus binding sequence based on the known experimentally determined Rns and AggR binding sequences^17^,^32^,^33^ (Fig. 3B) was generated with MEME and then submitted to FIMO^34^ for scanning the occurrence of motifs with p < 0.0001 in NC_018666.1.

### 5′ RACE and 3′ RACE

5′ RACE and 3′ RACE analyses were performed as described previously^21^. A detailed description is available in Supplementary Methods and the sequences of the RNA linker and primers used are given in Supplementary Table S5.

### Heterologous expression of SepA and AggR in *E. coli* K-12

Briefly, the *sepA* gene was cloned under the control of its native promoter in pBAD24 vector, whereas aggR was cloned downstream of the arabinose inducible promoter in pBAD33. The plasmids were then transformed in *E. coli* K-12 strain CSH50. For further details, please see Supplementary Methods.

### Quantification of SepA expression by western blot analysis

Briefly, for the SepA detection culture supernatants were separated by sodium dodecyl sulfate gel electrophoresis (SDS-PAGE) using Any kD Mini-Protean TGX precast gels (Bio-Rad) and transferred to a PVDF (polyvinylidenedifluoride) membrane using the Trans-Blot Turbo Transfer System (Bio-Rad). The rabbit antibody against SepA was synthesized by Aptum Biologics Ltd., UK. A secondary anti-Rabbit-HRP antibody (Dianova) was used. Chemiluminescent detection was performed using Clarity^TM^ ECL Western Blotting Substrate (Bio-Rad). Western blots were scanned with Chemidoc System (Bio-Rad) and the signal intensities of the bands of interests were quantified using the Image Lab Software version 5.0 (Bio-Rad). Dot plot diagrams summarizing the results from three biological replicates were generated using Python version 3.4. For further details, please refer to Supplementary Methods.

## Additional Information

**How to cite this article**: Berger, P. *et al.* The primary transcriptome of the *Escherichia coli* O104:H4 pAA plasmid and novel insights into its virulence gene expression and regulation. *Sci. Rep.* **6**, 35307; doi: 10.1038/srep35307 (2016).

## Supplementary Material

Supplementary Information

Supplementary Dataset1

Supplementary Dataset2
