# Supplementary material for: The primary transcriptome of the Escherichia coli O104:H4 pAA plasmid and novel insights into its virulence gene expression and regulation
Source: Sci Rep. 2016 Oct 17;6:35307. doi: 10.1038/srep35307 (PMC5066232; doi:10.1038/srep35307)
Supplement: Supplementary Information [file srep35307-s1.pdf]

The primary transcriptome of the *Escherichia coli* O104:H4 pAA plasmid and novel insights into its virulence gene expression and regulation

Petya Berger[a,*,+], Michael Knödler[a,+], Konrad U. Förstner[b,c,d,+], Michael Berger[a], Christian Bertling[a], Cynthia M. Sharma[d], Jörg Vogel[c], Helge Karch[a], Ulrich Dobrindt[a], Alexander Mellmann[a]

Institute of Hygiene, University of Münster, Münster, Germany[a]; Core Unit Systems Medicine, University of Würzburg, Würzburg, Germany[b]; Institute for Molecular Infection Biology, University of Würzburg, Würzburg, Germany[c]; Research Center for Infectious Diseases, University of Würzburg, Würzburg, Germany[d]

*corresponding author: Petya Berger, petya.berger@ukmuenster.de

[+]these authors contributed equally to this work

Supplementary Material

Supplementary Methods

Depletion of processed RNAs

Depletion of processed RNAs was performed by vertis Biotechnology AG, Germany using Terminator 5′-Phosphate-Dependent Exonuclease (TEX, Epicentre) as described previously[1]. Briefly, gDNA-free total RNA was treated with TEX (1U per µg of RNA) in the presence of RNase Inhibitor for 60 min at 30°C. A control reaction without TEX was performed in parallel. Following organic extraction, RNA was recovered by overnight precipitation and resuspended in RNase-free water.

cDNA libraby construction

cDNA libraries for the Illumina sequencing platform were constructed by vertis Biotechnology AG, Germany, as described previously[2], without the RNA size-fractionation step prior to cDNA synthesis. Briefly, equal amounts of TEX treated and untreated RNA were poly(A)-tailed using poly(A) polymerase. The samples were further treated with tobacco acid pyrophosphatase (TAP, Epicentre), which converts 5'-PPP to 5'-P, and thus prepared for the subsequent ligation of the 5’ end RNA adapter. First-strand cDNA was synthesized using an oligo(dT)-adapter primer and the M-MLV reverse transcriptase. The cDNA concentration was increased to 20-30 ng/µl in a PCR-based amplification step using a high fidelity DNA polymerase. The following adapter sequences flank the cDNA inserts:

TrueSeq Sense primer:

5'-AATGATACGGCGACCACCGAGATCTACACTCTTTCCCTACACGACGCTCTTCC
GATCT-3'

TrueSeq Antisense NNNNNN primer (NNNNNN = a library-specific barcode for multiplex sequencing):

5'-CAAGCAGAAGACGGCATACGAGAT-NNNNNN-GTGACTGGAGTTCAGACGTGT GCTCTTCCGATC(dT25)-3'

Read mapping of raw sequence reads

Raw reads in FASTQ format (approx. 7.2 Mio reads from each library) were trimmed with a cut-off Phred score of 20 and converted to FASTA format using the program fastq_quality_trimmer and fastq_to_fasta of the FASTX toolkit version 0.0.13 (http://hannonlab.cshl.edu/fastx_toolkit/). Sequences of rRNAs and tRNAs were extracted from the *E. coli* O104:H4 chromosome (NCBI Accession number NC_018658.1) and used as reference sequences for the read mapping with READemption version 0.3.7[3]and segemehl version 0.2.0[4], which included adaptor and poly(A)-trimming Reads that could not be mapped in this step (non rRNA and tRNA reads, 3.5 and 6.5 Mio reads for the TEX+ and TEX- library, respectively) were further used and mapped against the complete *E. coli* O104:H4 sequence including the chromosome and the plasmids pESBL, pG and pAA (NC_018658.1, NC_018659.1., NC_018660.1, and NC_018666.1, respectively). The NC_018666.1 file depicting the pAA gene annotation was updated based on our re-annotation (see “Reannotation of NC_018666 and operon organization of pAA”) and used in the mapping and further analysis. In total, 54,408 and 109,883 reads were mapped against pAA in TEX+ and TEX- library, respectively. Based on the resulting alignments for each library, coverage graphs representing the number of mapped reads per nucleotide in pAA were calculated and normalized to the total number of *E. coli* O104:H4 reads mapped per library (3,336,990 and 6,098,067 in TEX+ and TEX-, respectively) using READemption. The coverage values of

non-unique reads (reads mapped to multiple regions) were corrected based on (divided by) the number of locations they could be mapped to.

TSS and PS annotation

Annotation of transcription start sites (TSS) and processing sites (PS) candidates was performed with the TSSPredator program available at http://it.inf.uni-tuebingen.de/TSSpredator[5]. Briefly, the coverage graphs for the TEX+ and TEX- libraries generated by READemtion were converted into GR files and served as input for TSSpredator. TSS and PS annotation was performed with modified sensitive parameters and by searching for 5' ends, which were enriched in TEX+ in comparison to TEX- and vice versa, respectively. For the exact parameters used, please refer to the Shell script available at https://zenodo.org/record/45489 (DOI:10.5281/zenodo.45489). In total, 248 TSS were annotated with stepHeight =/>3, stepFactor >1,25, enrichment =/>1.5 and 79 PS with stepHeight=/>5,48, stepFactor>1,25, enrichment =/>1.5. stepHeight is the expression height change at the 5' RNA end position defined by the number of cDNA reads starting at the respective position: (e(i) - e(i-1); e(i): expression height at position i), stepFactor is the factor of height change at the 5' RNA end position: (e(i)/e(i-1); e(i): expression height at position i), and enrichment is the enrichment factor at the 5' RNA end position. While the stepFactor and enrichment values were kept constant in the TSS and PS annotation, the difference in the stepHeight values resulted from the fold difference (1.83x) in the total number of *E. coli* O104:H4 reads mapped in TEX- in comparison to TEX+.

Stable prediction of the downstream regions of pAA-associated TSS

Minimum free energy secondary structures of the 50 and 100 nt downstream regions of pAA-associated TSS mapped by dRNA-seq were predicted using RNAfold (part of the Vienna RNA Package version 2.1.9[6]) with default parameters and “-p” flag to retrieve the partition function and base pairing probability matrix. Using Python version 3.4, mountain plot values representing the number of enclosing nucleotides per nucleotide position were calculated based on the minimum free energy structures and the mountain plot distributions were visualized as box plots.

Reannotation of NC_018666 and operon organization of pAA

There are 80 coding sequences (CDS) annotated in the pAA plasmid of *E. coli* O104:H4 (NC_018666.1[7]). Based on the operon annotation available in the Database of prokaryotic OpeRons (DOOR) 2.0[8], 45 of them are clustered in 18 operons and 35 are standing alone genes. A comparison with the pAA genes which are part of the AggR regulon in EAEC 042[9] revealed that two known/hypothetical virulence-associated genes are missing and one is wrongly annotated in the current pAA annotation of *E. coli* O104:H4. Therefore, we introduced the following changes in NC_018666 annotation: (i) *aar* coding for the AggR-activated regulator Aar[10] was annotated in the region encompassing nucleotide position 74181-164 on the minus strand. The *E. coli* O104:H4 homolgue is 3 amino acids longer in comparison to the one in EAEC 042; (ii) *aap* coding for dispersin[11] was annotated with the coordinates 2094-2444 on the minus strand; and (iii) O3K_26127, a homologue of the ECO42_pAA004 gene coding for a hypothetical protein in the EAEC strain 042 was extended from 11391-11678 to 11391-11930 on the minus strand and thus replaced O3K_26132 (11687-11911, minus strand), which in the DOOR operon annotation are part of a three gene

operon (O3K_26137-26132-26127). In addition, the following changes in the operon annotation were introduced: (i) The genes *aap* and *aar* are not mapped in the vicinity of any previously annotated gene in NC_018666 and were therefore assumed to be transcribed monocistronically; (ii) the AAF/I operon-encoding genes O3K_26212, 26207, 26202, 26197, were clustered in a single operon[12]; (iii) O3K_26192, considered to be co-transcribed with O3K_26197 (the last gene of the AAF/I cluster) in DOOR was changed to an alone standing gene based on the 3' end of the AAF/I operon mapped in this study. (iv) operons O3K_26482-26477 and O3K_26472-26467-26462 which encode the genes of the *aatPABCD* cluster[13] were combined into one transcription unit. In summary, we assume in our analysis that there are 81 coding sequences in *E. coli* O104:H4 pAA plasmid, 44 of them clustered in 16 operons and 37 transcribed as alone standing genes (Supplementary Table S2).

Correlation of AggR binding site occurrence and gene type

The correlation of predicted AggR binding sites with gene function was evaluated using the following information: the operon information described in the "Reannotation of NC_018666 and operon organization of pAA" section, gTSS and gPS (mapped within the 300 nt upstream regions of ORFs) annotated by TSSpredators (Supplementary Dataset S1 and S2) and the FIMO-predicted AggR binding sites (Supplementary Table S4). The FIMO hits were considered to be associated with genes when they were located in the region from - 250 nt in respect to detected mRNA end/ATG to + 50 nt in respect to ATG of monocistronic genes or first genes in an operon. The mRNA end coordinates were considered for genes with annotated gTSS or gPS (in case no gTSS could be detected) or ATG for genes with no detected gTSS or gPS. The coordinates used for the AggR analysis are based on the locations of the experimentally determined binding sites used for the generation of the AggR consensus binding motif[14-16]. Once a predicted transcription factor binding site was mapped within the

above described regions of a first gene of an operon, then all genes of this operon were considered to be associated with the corresponding binding site. The list of virulence genes was created based on published data[10,11,13,17-21]. The number of virulence gene with and without associated AggR/H-NS predicted binding sites were compared to the number of non-virulence genes with and without those biding site hits by means of a Fisher's exact test.

5’ RACE & 3’ RACE

TSS and PS candidates represented with various stepHeight (cDNA reads starting at the respective position) and enrichmentFactor (enrichment between differential libraries) values, and belonging to different categories were selected for verification in a biological replicate by 5’ RACE analysis. TSS and PS assigned to known or hypothetical virulence-associated genes were preferentially chosen. 5’ RACE and 3’ RACE analyses were performed as previously described[22]. For 5’ RACE 1µg of total RNA from LB226692 was treated with tobacco acid pyrophosphatase (TAP; Epicentre) or incubated in buffer alone for 60 min at 37 °C. Next, 5’ RNA linker (5R_L; Supplementary Table S5) was ligated to the RNA for 60 min at 37°C using 40 U of T4 RNA ligase (Epicentre). Reverse transcription was carried out with up to 6 target specific reverse primers and SuperScript III reverse transcriptase (LifeTechnologies). For verification of gTSS and aTSS, the target specific primers were placed within the ORFs to which the TSS were assigned. All above described reactions were purified by an organic extraction (25:24:1 v/v phenol/chloroform/isoamyalcohol) and RNA was recovered by overnight precipitation with 3 volumes of ethanol/3 M sodium acetate at -20°C. Nested PCRs were set with target and linker specific primers and the cDNA from the reverse transcription step as template. The mature 16S rRNA, which has a 5’-P end, was used as a 3’ linker in 3’ RACE. 1µg of total RNA from LB226692 was self-ligated for 60 min at 37°C using 40 U of T4 RNA ligase (Epicentre). Control reactions without ligase were included. Next, RNA was

reverse-transcribed using *rrn16* specific primer (3R_1). Target and *rrn16* specific primers were used in nested PCRs for the amplification of the reverse transcription products. 5'- and 3' RACE PCR products were analyzed on 1.5% agarose gels and products of interest were excised, purified and Sanger sequenced using BigDye® Terminator Cycle Sequencing Kit (Life Technologies). PCR products containing multiple 5'or 3' pAA termini were ligated into pGEM®-T (Promega) and transformed into NEB 5-alpha competent *E.coli* cells (New England Biolabs). 5 bacterial clones per transformation were selected, their DNA was PCR amplified with primers M13_for and rev (vector specific primer) and sequenced as described above. To ensure that the detected 5' ends in 5' RACE are not amplification artifacts, only different nucleotide combination at the NNN region of the RNA linker were considered for the calculation of the number of clones supporting dRNA-seq data (Supplementary Table S1).

Heterologous expression of SepA and AggR in *E. coli* K-12

The pAA regions from position 55353 to 59821, encompassing *sepA*, its promoter region and both predicted AggR binding sites, and from position 6823 to 7773, containing *aggR* without the promoter region were amplified from total DNA using the Phusion Polymerase (Thermo Scientific) and the primers PB74 + PB75 and PB76 + PB77, respectively. The fragment containing *sepA* was digested with XbaI (restriction site was introduced in the 5' end of the reverse primer PB75) and ligated into pBAD24[23] linearized with the restriction enzymes XbaI and EcoRV. This cloning strategy allowed for the expression of SepA under the control of its native promoter. KpnI and XbaI sites were added to the 5' ends of the forward (PB76) and reverse (PB77) primer, respectively, used for *aggR* amplification. After digestion with the respective enzymes, the *aggR*-containing fragment was ligated into the corresponding sites in pBAD33[23] and it was thus cloned under control of the arabinose-inducible promoter. Sanger sequencing was used to verify the correct sequence of the cloned constructs. The plasmids

pSepA carrying *sepA* with its native promoter was later on used as a template for a PCR with primers PB76 and PB77. The obtained product was digested with XbaI and ligated into pBAD24[23] linearized with the restriction enzymes XbaI and EcoRV. This cloning strategy allowed for the construction of the plasmid pSepA* carrying the pAA region from position 55507 to 59821, encompassing *sepA* and its native promoter region but excluding the upstream predicted AggR binding sites. The correct sequence of the cloned construct was verified by Sanger sequencing. The plasmids carrying *sepA* with its native promoter and the predicted AggR binding sites (pSepA; Amp^R), *sepA* with its native promoter and excluding the upstream predicted AggR binding sites (pSepA*; Amp^R), *aggR* under the transcriptional control of an inducible promoter (pBAD33-AggR, Cm^R), pBAD24 (Amp^R) and pBAD33 (Cm^R) were transformed in electrocompetent cells of the *E. coli* K-12 strain CSH50[24].

Analysis of SepA expression by semi-quantitative Western blot

The *E. coli* O104:H4 strain C227-11Φcu cured of Stx2a encoding phage[25,26] and *E. coli* K-12 strain CSH50 carrying the plasmids pBAD24, pSepA pBAD33, pSepA pBAD33-AggR, pSepA* pBAD33 and pSepA* pBAD33-AggR were used for the validation of the AggR-dependent SepA expression by Western blot analysis. Single colonies were inoculated in 2 mL LB medium alone (for *E. coli* O104:H4 strain C227-11Φcu), LB supplemented with 100µg/mL Amp (for CSH50 pBAD24) or with 100µg/mL Amp + 25µg/mL Cm (for the rest) and grown overnight at 37°C, 180rpm. The overnight cultures were diluted 1:1,000 in LB medium or LB medium supplemented with 1% arabinose and the corresponding antibiotics. Bacteria were grown for 16 h at 37°C, 180rpm and the culture supernatants were retrieved by centrifugation for 20 min at 4°C, 3000g. The culture supernatant of the *E. coli* O104:H4 strain C227-11Φcu was 10x concentrated using a Vivaspin 20 concentrator with molecular cut-off 3,000 (GE Healthcare). The volumes of the rest of the culture supernatant were normalized to

final OD_{600} of the overnight cultures. The culture supernatants were stored at $4^{o}C$ for the subsequent SepA detection.

Twelve µL of the culture supernatants were separated by sodium dodecyl sulfate gel electrophoresis (SDS-PAGE) using Any kD Mini-Protean TGX precast gels (Bio-Rad), and transferred to a PVDF (polyvinylidenedifluoride) membrane (Bio-Rad) using the Trans-Blot Turbo Transfer System (Bio-Rad). SDS-PAGE and blotting were performed according to the Bio-Rad instruction manual. The peptide antibody used for SepA detection was synthesized by Aptum Biologics Ltd, UK. Membranes were blocked in TBS-T (TBS containing 0.05 % Tween 20) and 5 % (w/v) milk powder overnight at 4 ^{o}C, incubated with the SepA antibody (1:100 diluted in TBS-T) for 2 h and a secondary anti-Rabbit-HRP antibody (Dianova) for 2 h at room temperature. Chemiluminescence detection was performed using the Clarity^{TM} ECL Western Blotting Substrate (Bio-Rad).

Western blots were scanned with the Chemidoc System (Bio-Rad) and the signal intensities of the bands of interests were quantified using the Lane and Bands Tools of the Image Lab Software Version 5.0 (Bio-Rad). For the final quantification, the samples of three biological replicates were analysed from a single immunoblot membrane and the quantified signal intensity (computer internal units) were used to determine differences in SepA and AggA expression. Dot plot diagrams depicting the quantified signal intensity were generated using Python version 3.4.

References

1 Sharma, C. M. *et al.* The primary transcriptome of the major human pathogen *Helicobacter pylori*. *Nature* **464**, 250-255, (2010).

2 Berezikov, E. *et al.* Diversity of microRNAs in human and chimpanzee brain. *Nat. Genet.* **38**, 1375-1377, (2006).

3 Förstner, K. U., Vogel, J. & Sharma, C. M. M. READemption - A tool for the computational analysis of deep-sequencing-based transcriptome data. *Bioinformatics* **doi: 10.1093/bioinformatics/btu533**, (2014).

4 Hoffmann, S. *et al.* Fast mapping of short sequences with mismatches, insertions and deletions using index structures. *PLoS Comput. Biol.* **5**, e1000502, (2009).

5 Dugar, G. *et al.* High-resolution transcriptome maps reveal strain-specific regulatory features of multiple *Campylobacter jejuni* isolates. *PLoS Genet.* **9**, e1003495, (2013).

6 Lorenz, R. *et al.* ViennaRNA Package 2.0. *Algorithms Mol. Biol.* **6**, 26, (2011).

7 Ahmed, S. A. *et al.* Genomic comparison of *Escherichia coli* O104:H4 isolates from 2009 and 2011 reveals plasmid, and prophage heterogeneity, including Shiga toxin encoding phage *stx2*. *PLoS One* **7**, e48228, (2012).

8 Mao, F., Dam, P., Chou, J., Olman, V. & Xu, Y. DOOR: a database for prokaryotic operons. *Nucleic Acids Res.* **37**, D459-463, (2009).

9 Morin, N., Santiago, A. E., Ernst, R. K., Guillot, S. J. & Nataro, J. P. Characterization of the AggR regulon in enteroaggregative *Escherichia coli*. *Infect. Immun.* **81**, 122-132, (2013).

10 Santiago, A. E. *et al.* A large family of antivirulence regulators modulates the effects of transcriptional activators in Gram-negative pathogenic bacteria. *PLoS Path.* **10**, e1004153, (2014).

11 Sheikh, J. *et al.* A novel dispersin protein in enteroaggregative *Escherichia coli*. *J. Clin. Invest.* **110**, 1329-1337, (2002).

12 Nataro, J. P. *et al.* Aggregative adherence fimbria I expression in enteroaggregative *Escherichia coli* requires two unlinked plasmid regions. *Infect. Immun.* **61**, 1126-1131, (1993).

13 Nishi, J. *et al.* The export of coat protein from enteroaggregative *Escherichia coli* by a specific ATP-binding cassette transporter system. *J. Biol. Chem.* **278**, 45680-45689, (2003).

14 Morin, N. *et al.* Autoactivation of the AggR regulator of enteroaggregative *Escherichia coli in vitro* and *in vivo*. *FEMS Immunol. Med. Microbiol.* **58**, 344-355, (2010).

15 Munson, G. P. & Scott, J. R. Binding site recognition by Rns, a virulence regulator in the AraC family. *J. Bacteriol.* **181**, 2110-2117, (1999).

16 Munson, G. P., Holcomb, L. G. & Scott, J. R. Novel group of virulence activators within the AraC family that are not restricted to upstream binding sites. *Infect. Immun.* **69**, 186-193, (2001).

17 Bielaszewska, M. *et al.* Characterisation of the *Escherichia coli* strain associated with an outbreak of haemolytic uraemic syndrome in Germany, 2011: a microbiological study. *Lancet Infect. Dis.* **11**, 671-676, (2011).

18 Mellmann, A. *et al.* Prospective genomic characterization of the German enterohemorrhagic *Escherichia coli* O104:H4 outbreak by rapid next generation sequencing technology. *PLoS One* **6**, e22751, (2011).

19 Rasko, D. A. *et al.* Origins of the *E. coli* strain causing an outbreak of hemolytic-uremic syndrome in Germany. *New Engl. J. Med.* **365**, 709-717, (2011).

20 Brzuszkiewicz, E. *et al.* Genome sequence analyses of two isolates from the recent *Escherichia coli* outbreak in Germany reveal the emergence of a new pathotype: Entero-Aggregative-Haemorrhagic *Escherichia coli* (EAHEC). *Arch. Microbiol.* **193**, 883-891, (2011).

21 Boisen, N. *et al.* The presence of the pAA plasmid in the German O104:H4 Shiga toxin type 2a (Stx2a)-producing enteroaggregative *Escherichia coli* strain promotes the translocation of Stx2a across an epithelial cell monolayer. *J. Infect. Dis.* **210**, 1909-1919, (2014).

22 Zhelyazkova, P. *et al.* The primary transcriptome of barley chloroplasts: numerous noncoding RNAs and the dominating role of the plastid-encoded RNA polymerase. *Plant Cell* **24**, 123-136, (2012).

23 Guzman, L. M., Belin, D., Carson, M. J. & Beckwith, J. Tight regulation, modulation, and high-level expression by vectors containing the arabinose PBAD promoter. *J. Bacteriol.* **177**, 4121-4130, (1995).

24 Miller , J. H. *Experiments in Molecular Genetics*. (Cold Spring Harbor Laboratory, 1972).

25 Zangari, T. *et al.* Virulence of the Shiga toxin type 2-expressing *Escherichia coli* O104:H4 German outbreak isolate in two animal models. *Infect. Immun.* **81**, 1562-1574, (2013).
26 Kunsmann, L. *et al.* Virulence from vesicles: Novel mechanisms of host cell injury by *Escherichia coli* O104:H4 outbreak strain. *Sci. Rep.* **5**, 13252, (2015).

Supplementary Table S1. 5'-RACE verification of pAA-associated TSS and PS mapped by dRNA-seq. TSS and PS belonging to different categories and characterized with various abundance (stepHeight) and enrichment (enrichmentFactor) were subjected to 5'-RACE verification. The number of clones from TAP+ and/or TAP- 5'-RACE reactions supporting the dRNA-seq data are listed (1/1 = PCR product contained a single 5' end and did not require subsequent cloning). The nucleotide positions of the additional TSS/PS mapped by 5'-RACE analysis and the supporting data are also included.

		dRNA-seq						5'-RACE					Comments
								Confirmation of dRNA-seq		Additional 5' end			
		Position	Strand	step Height[a]	enrichment Factor	TSS/PS category	Locus tag (Gene name)	TAP+[b]	TAP-[b]	Position	TAP+[b]	TAP-[b]	
**TSS**	1	925	-	3,48	3,03	aTSS	O3K_26057	4/4	-				
	2	1994	+	6	2,15	gTSS	(*aap*)	3/3	-				
	3	1987	+	13	1,94	gTSS	(*aap*)	0/3	-				
	4	2433	-	3338	2,35	aTSS	(*aap*)	3/5	-				
	5	9633	+	4,57	2,72	aTSS	O3K_26112	1/1	-				
	6	9764	+	3,48	2,78	aTSS	O3K_26112	4/4	-				
	7	10680	+	14	1,64	gTSS	O3K_26122	2/3	1/5	10690	0/3	3/5	
	8					iTSS	O3K_26117						
	9	12275	-	27	3,75	ITSS	O3K_26137	3/4	-				Detected as a gTSS of O3K_26127
	10	13268	-	25	5,71	oTSS		2/3	0/1				Detected as a gTSS of O3K_26137
	11	17351	+	3	7,31	gTSS	O3K_26187	3/3					
	12	19566	-	68	2,54	iTSS	O3K_26207 (*aggC*)	1/1	-				Detected as a gTSS of *aggB*
	13	20268	-	97	1,6	iTSS	O3K_26207 (*aggC*)	1/1	-				Detected as a gTSS of *aggB*
	14	22022	-	4	7,31	iTSS	O3K_26212 (*aggD*)	3/3	-				
	15	26234	+	164	3,5	aTSS	O3K_26242 (*parM*)	1/1	-				
	16	30158	-	15	2,35	gTSS	O3K_26267 (*ccdA*)	1/3	-				
	17	39430	-	3,48	3,03	aTSS	O3K_26337	4/4	-				
	18	39561	-	4,24	3	aTSS	O3K_26337	1/1	-				
	19	44859	-	17	3,88	iTSS	O3K_26382 (*traX*)	4/4	-				
	20					gTSS	O3K_26377 (*finO*)						
	21	55560	+	5	1,66	gTSS	O3K_26432 (*sepA*)	3/5	-				
	22	66404	-	4	7,31	gTSS	O3K_26462 (*aatD*)	4/5	-				
	23	67507	-	15	6,85	gTSS	O3K_26472 (*aatB*)	1/2	-				
	24	73951	+	10	3,05	oTSS		0/4					
	25	73955	+	51	6,88	oTSS		4/4	-				
	26	74127	-	438	2,87	oTSS		4/4	-				
**PS**	1	195	-	12,59	>100	gPS	*aap*	-	3/5	148	-	2/5	
	2	7674	+	38,31	2,02	gPS	O3K_26102	1/1	1/1				
	3	19439	-	51,44	1,84	iPS	O3K_26207 (*aggC*)	1/1	-				Detected as a TSS; Detected as a gTSS of *aggB*
	4	22460	-	5,64	6,31	gPS	O3K_26212 (*aggD*)	1/3	2/3				
	5	29961	-	110,54	2,27	gPS	O3K_26267 (*ccdA*)	1/1	1/1				
	6	38370	-	151,58	2	gPS	O3K_26332 (*repA2*)	2/5	0/4	38358	2/5	4/4	Detected as a TSS
	7	44802	-	24,62	1,53	gPS	O3K_26377 (*finO*)	4/4	2/4	44777	0/4	2/4	Detected as a TSS
	8	55691	+	74,7	1,92	iPS	O3K_26432 (*sepA*)	-	3/6	55640/55641	-	2/6	
	9	59728	+	633,13	1,96	iPS	O3K_26432 (*sepA*)	3/3	4/4				
	10	66465	+	14,77	1,86	aPS	O3K_26467 (*aatC*)	2/2	1/1				

[a]The expression height change at the 5' RNA end position defined by the number of cDNA reads starting at the respective position: (e(i) - e(i-1); e(i): expression height at position i).
[b]The number of clones in the TAP+/TAP- reaction suporting the dRNA-seq data: x/y; x: number of clones in agreement with dRNA-seq results; y: total number of sequenced clones; 1/1 PCR product contained single 5' end and did not require cloning, - not sequenced.

Supplementary Table S2. Operon map of the pAA plasmid. The pAA operon organization and 5' mRNA ends detected by dRNA-seq are presented. The operon organization is based on the information available in the Database of prokaryotic operons (DOOR) and our re-annotation (see Supplementary Materials and Methods). Genes clustered in an operon are color-grouped and marked with 1 in the "Operon" column. The position of the candidate 5'-PPP and 5'-P mRNA ends, i.e. gTSS and gPS, is given. gTSS found internal to operons which could potentially be involved in its transcriptional uncoupling from the main upstream promoter are marked in red.

Locus tag (Gene name)	Strand	Gene start	Gene end	Operon	Monocistronic gene	5' mRNA end	
						gTSS (5'-PPP)	gPS (5'-P)
(*aar*)	-	74181	164		1	-	195
O3K_26057	-	757	1737		1	1768, 1764	-
O3K_26062	+	1736	1870		1	-	-
(*aap*)	+	2094	2444		1	1987, 1994	-
O3K_26077	-	3211	4734		1	-	-
O3K_26087	-	5805	6131		1	6184, 6338, 6303	-
O3K_26097 (*aggR*)	+	6865	7662		1	6739, 6649	-
O3K_26102	+	7763	8167		1	-	7674
O3K_26107	-	8410	9234		1	9274	-
O3K_26112	-	9596	10576		1	10603	-
O3K_26117	+	10575	10736	1		-	-
O3K_26122	+	10736	10888			**10680**	-
O3K_26127	-	11391	11930	1		-	-
O3K_26137	-	11934	12962			-	-
O3K_26147	+	13398	13898		1	13299, 13166	-
O3K_26152	+	14141	14329		1	14079	-
O3K_26157	-	14354	14578		1	14819	-
O3K_26162	+	14623	14880		1	-	-
O3K_26177	-	16503	16850	1		-	17067
O3K_26182	-	16847	17248			-	17260
O3K_26187	+	17434	17595		1	17247, 17351	-
O3K_26192	-	17564	17884		1	-	-

Locus tag (Gene name)	Strand	Gene start	Gene end	Operon	Monocistronic gene	5' mRNA end	
						gTSS (5'-PPP)	gPS (5'-P)
O3K_26197 (*aggA*)	-	18021	18524	1		-	18808
O3K_26202 (*aggB*)	-	18626	19063			-	-
O3K_26207 (*aggC*)	-	19077	21461			-	-
O3K_26212 (*aggD*)	-	21619	22248			-	22460
O3K_26217	-	22524	22727		1	-	-
O3K_26222	-	23226	23837		1	-	-
O3K_26227	+	23924	24301	1		23809, 23718	-
O3K_26232	+	24301	25497			-	-
O3K_26237 (*parR*)	-	25577	25993	1		**26158**	-
O3K_26242 (*parM*)	-	25986	26966			27027	-
O3K_26247	-	27380	27688		1	-	27737
O3K_26252	-	27775	28419		1	-	-
O3K_26257 (*repD*)	-	28598	29404	1		**29599**	-
O3K_26262 (*ccdB*)	-	29405	29710			-	29720
O3K_26267 (*ccdA*)	-	29712	29930			30158	29961
O3K_26272	+	30525	30755	1		-	30476
O3K_26277	+	30752	31180			-	-
O3K_26282	-	31263	31418		1	31661, 31594, 31550	-
O3K_26287	+	32504	33481		1	32347	-
O3K_26292	-	33760	34500		1	-	34538
O3K_26297	-	34869	34955		1	-	35026
O3K_26302	-	35232	35477	1		-	-
O3K_26307	-	35483	35758			-	-
O3K_26312	-	35758	36042			-	36061
O3K_26317	-	36950	37621	1		-	-
O3K_26322	-	37590	37778			-	-
O3K_26327 (*repL*)	-	37771	37845			38030	37902, 38037, 37924, 37874, 38075, 37880
O3K_26332 (*repA2*)	-	38083	38337		1	38370, 38608	
O3K_26337	+	38618	39598		1	38587, 38591	38482, 38492

Locus tag (Gene name)	Strand	Gene start	Gene end	Operon	Monocistronic gene	5' mRNA end	
						gTSS (5'-PPP)	gPS (5'-P)
O3K_26342	-	40006	40176		1	40313, 40376	-
O3K_26347	+	40326	41003	1		-	-
O3K_26352	+	41003	41350	1		-	-
O3K_26357	+	41370	42941	1		-	41370
O3K_26362	-	42995	43180	1		-	-
O3K_26367	-	43254	43550	1		**43610**	-
O3K_26372	-	43547	43981	1		-	-
O3K_26377 (*finO*)	-	44210	44770	1		**44859**	
O3K_26382 (*traX*)	-	44825	45571	1		-	-
O3K_26387	-	45591	45728	1		-	-
O3K_26392 (*traI*)	-	45804	49517	1		-	-
O3K_26397	-	49522	50196	1		-	-
O3K_26402	+	50318	50617		1	50302	-
O3K_26412 (*traM*)	-	51661	52044		1	52153	-
O3K_26417	+	52468	52977		1	-	-
O3K_26422	-	53295	54095		1	-	54321
O3K_26427	+	54890	55147		1	54724	-
O3K_26432 (*sepA*)	+	55669	59763		1	55560	-
O3K_26437	-	60058	60243		1	60263, 60252	-
O3K_26442	+	60357	60791	1		-	-
O3K_26447	+	60788	61138	1		**60728**	-
O3K_26452	+	61169	62668	1		**61093, 61169**	-
O3K_26457	-	62938	63978		1	-	-
O3K_26462 (*aatD*)	-	64953	66164	1		**66404**	-
O3K_26467 (*aatC*)	-	66181	66810	1		-	-
O3K_26472 (*aatB*)	-	66803	67441	1		**67507**	-
O3K_26477 (*aatA*)	-	67521	68759	1		-	-
O3K_26482 (*aatP*)	-	68756	69886	1		70088	-
O3K_26492	+	70508	70600		1	70361, 70218	-
O3K_26497	+	70657	70893		1	70606	-

Supplementary Table S3. pAA-associated antisense and non-coding RNAs candidates subjected to 3' RACE. The 3' ends of selected antisense RNA (associated with an aTSS) and non-coding RNA candidates (associated with an oTSS) were determined by 3' RACE and the size (nt) of the transcripts was calculated. The predominantly detected 3' ends and lengths are marked in bold.

<table>
<tr><th></th><th colspan="7">dRNA-seq</th><th colspan="2">3' RACE</th></tr>
<tr><th></th><th>5' end position</th><th>Strand</th><th>step Height</th><th>step Factor</th><th>enrichment Factor</th><th>TSS category</th><th>Locus tag (gene name)</th><th>3' end position</th><th>RNA Size (nt)</th></tr>
<tr><td>1</td><td>2433</td><td>-</td><td>3338</td><td>6,6</td><td>2,35</td><td>aTSS</td><td>(aap)</td><td>2342-2343</td><td>91-92</td></tr>
<tr><td rowspan="2">2</td><td rowspan="2">6357</td><td rowspan="2">+</td><td rowspan="2">553,83</td><td rowspan="2">3,56</td><td rowspan="2">1,5</td><td rowspan="2">oTSS</td><td rowspan="2"></td><td>6452, 6472, 6475</td><td>95, 116, 118</td></tr>
<tr><td>6604, 6618</td><td>247, 261</td></tr>
<tr><td>3</td><td>45870</td><td>+</td><td>5</td><td>>100</td><td>2,28</td><td>aTSS</td><td>O3K_26392 (traI)</td><td>45944-45947</td><td>74-77</td></tr>
<tr><td rowspan="2">4</td><td rowspan="2">57462</td><td rowspan="2">-</td><td rowspan="2">19</td><td rowspan="2">2,46</td><td rowspan="2">9,75</td><td rowspan="2">aTSS</td><td rowspan="2">O3K_26432 (sepA)</td><td>57402</td><td>60</td></tr>
<tr><td>57315, 57346</td><td>116, 147</td></tr>
<tr><td>5</td><td>59447</td><td>-</td><td>12</td><td>4</td><td>3,25</td><td>aTSS</td><td>O3K_26432 (sepA)</td><td>59348, 59364, 59375, 59392</td><td>55, 72, 83, 99</td></tr>
<tr><td>6</td><td>59885</td><td>-</td><td>108</td><td>16,43</td><td>1,75</td><td>oTSS</td><td></td><td>59768-59770</td><td>117-115</td></tr>
<tr><td rowspan="2">7</td><td>73951</td><td>+</td><td>10</td><td>>100</td><td>3,05</td><td rowspan="2">oTSS</td><td rowspan="2"></td><td rowspan="2">74066, 74022</td><td rowspan="2">67/71, 111/115</td></tr>
<tr><td>73955</td><td>+</td><td>51</td><td>4,92</td><td>6,88</td></tr>
</table>

Supplementary Table S4. Predicted AggR binding sites in the pAA plasmid. AggR binding sites were computationally predicted in pAA using FIMO from the MEME suite (http://meme-suite.org/tools/fimo). Predicted binding sites with a p-value < 0.0001 are listed and ranked by increasing p-value. FIMO hits considered to be associated with *E. coli* O104:H4 virulence genes or homologues of the genes part of the AggR regulon in EAEC strain 042 (see Supplementary Material and Methods) are marked in bold and the position of the motifs in respect to gTSS/gPS/ATG is given in "Comments".

Motif Ranking[a]	Strand	Start	End	p-value[b]	q-value[c]	Sequence	Comments
**1**	**+**	**6779**	**6798**	**2.15e-08**	**0.00306**	**TTAAAAATTTATCTTTTTAT**	**130 nt downstream of *aggR* gTSS_6649**
2	+	72822	72841	6.92e-08	0.00306	TTATTTTTTTATCATTTTGA	
**3**	**-**	**55487**	**55506**	**6.92e-08**	**0.00306**	**TTATTTTTTTATCATTTTGA**	**54 nt upstream *sepA* gTSS_55560**
**4**	**-**	**6855**	**6874**	**8.29e-08**	**0.00306**	**TTAATTTCATATCATTCTCA**	**overlaps with *aggR* gene start (6865)**
**5**	**+**	**10667**	**10686**	**3,00E-06**	**0.0886**	**CTATAATAATATCTATTTTT**	**overlaps with O3K_26122 gTSS_10680**
**6**	**-**	**1883**	**1902**	**4.67e-06**	**0.115**	**CTATTTTTATATTATTTTAT**	**85 nt upstream *aap* gTSS_1987**
7	-	11948	11967	6.41e-06	0.135	TGAATTACATTTTTTTCTGT	
8	-	64438	64457	8.12e-06	0.15	TAATTTTATAATCTCTTTTT	
**9**	**+**	**22587**	**22606**	**9.26e-06**	**0.15**	**ATAATTATTTATCACGTTAT**	**127 nt upstream *aggD* gPS_22460**
10	+	18128	18147	1.02e-05	0.15	TCAATTTTTTATAAATTTTT	
11	+	67841	67860	1.15e-05	0.155	AAATAATCTTTTCTTTTTGA	
12	+	68343	68362	1.49e-05	0.183	AGATTTTTTTATCTTATAGT	
**13**	**+**	**69990**	**70009**	**1.73e-05**	**0.191**	**ATATATAATTATAAGTTTAT**	**79 nt downstream of *aatP* gTSS_70088**
14	+	26612	26631	2.25e-05	0.191	TATTTTTCTTATTTTTCTGA	
15	-	30359	30378	2.27e-05	0.191	ACATATCTATATTTCTTTCT	
**16**	**-**	**69984**	**70003**	**2.28e-05**	**0.191**	**TTATAATTATATATCCCTTA**	**85 nt downstream of *aatP* gTSS_70088**
**17**	**-**	**67552**	**67571**	**2.29e-05**	**0.191**	**TTATATCATTATTTTCTTCT**	**45 nt usptream *aatB* gTSS_67507**
**18**	**-**	**410**	**429**	**2.33e-05**	**0.191**	**ATATTAATATATCAAATGTA**	**215 nt upstream *aar* gPS_195**
19	-	64565	64584	2.63e-05	0.192	ATATATTAACATCTCTCTCA	
20	+	52057	52076	2.69e-05	0.192	TTATTTTCTTATCGCATCAA	
21	-	38469	38488	2.88e-05	0.192	TCATTTTATTATCAAAATTA	
22	+	69437	69456	3.1e-05	0.192	ATTAATAAATATTTGTTTCT	
23	+	12296	12315	3.25e-05	0.192	TCATCATATTATCAAACTGA	
24	-	11513	11532	3.25e-05	0.192	TTATAAATATGTTTCCTTGA	
25	+	21428	21447	3.54e-05	0.192	TTAAAATTATATCAACCGGA	
26	-	12046	12065	3.7e-05	0.192	AAAAAAATAAATCATCCTCT	
27	+	23253	23272	3.87e-05	0.192	ATCATTTTTTATTGTTCTTT	
28	-	18567	18586	3.96e-05	0.192	TAAATAATATCTTGATCTAT	
29	-	10768	10787	4.24e-05	0.192	TGATTATATTATCAAAATCA	

Motif Ranking[a]	Strand	Start	End	p-value[b]	q-value[c]	Sequence	Comments
30	-	7019	7038	4.29e-05	0.192	TGATATATTTATTCCTCTCT	
31	+	69240	69259	4.33e-05	0.192	TTGTTTATATCTCTCTTTGT	
32	-	65739	65758	4.34e-05	0.192	GTCTAAAAATATCATTTTTT	
33	+	35057	35076	4.37e-05	0.192	TAATATATTTATCAGCCTAC	
34	-	30365	30384	4.44e-05	0.192	TGTTTAACATATCTATATTT	
35	-	52314	52333	4.65e-05	0.195	TCATTAACTTATGTTTTAAA	
36	-	30357	30376	5.03e-05	0.195	ATATCTATATTTCTTTCTTT	
37	-	65830	65849	5.03e-05	0.195	TTATATTATGATTTTCCTGA	
38	-	35051	35070	5.16e-05	0.195	TGATAAATATATTAATCAGA	
39	+	30488	30507	5.17e-05	0.195	TGATTCATATATCCATCTGT	
**40**	**-**	**69992**	**70011**	**5.39e-05**	**0.196**	**TCATAAACTTATAATTATAT**	**77 nt downstream of *aatP* gTSS_70088**
41	-	21610	21629	5.44e-05	0.196	TTAATATTTAATCATGTTTT	
**42**	**-**	**69982**	**70001**	**5.66e-05**	**0.199**	**ATAATTATATATCCCTTAGT**	**87 nt downstream of *aatP* gTSS_70088**
**43**	**-**	**13000**	**13019**	**5.88e-05**	**0.199**	**AAAATAATTTTTTGGTTTTT**	**38 nt upstream O3K_26137 gene start (12962)**
**44**	**+**	**67582**	**67601**	**6.04e-05**	**0.199**	**TCAATCTCATATCCAATTTT**	**75 nt usptream *aatB* gTSS_67507**
45	-	25407	25426	6.08e-05	0.199	TCATTAACTTTTCACTGTTT	
46	-	68482	68501	6.38e-05	0.204	TCATTATCCTATAATCTTCT	
**47**	**-**	**13055**	**13074**	**6.55e-05**	**0.206**	**CTATAATAATATCTATTATT**	**93 nt upstream O3K_26137 gene start (12962)**
48	-	66978	66997	6.98e-05	0.215	AGAATAAGGTATCGTTCTTT	
**49**	**-**	**22579**	**22598**	**7.22e-05**	**0.217**	**ATAAATAATTATCTTTATGC**	**119 nt upstream *aggD* gPS_22460**
50	-	22134	22153	7.4e-05	0.218	TCAAAAAAATATTGCACCTT	
**51**	**+**	**55672**	**55691**	**7.89e-05**	**0.227**	**AACAAAATATATTATCTTAA**	**112 nt downstream of *sepA* gTSS_55560**
**52**	**-**	**6686**	**6705**	**7.99e-05**	**0.227**	**GCAATTATTTATCGTGTTCT**	**37 nt downstream of *aggR* gTSS_6649**
53	-	27174	27193	8.33e-05	0.227	ATGTTTTTATATTGCATTTA	
54	-	65594	65613	8.33e-05	0.227	TAATTTTTATACTGATCTCA	
**55**	**-**	**13053**	**13072**	**8.72e-05**	**0.234**	**ATAATAATATCTATTATTTT**	**91 nt upstream O3K_26137 gene start (12962)**
56	+	68200	68219	9.19e-05	0.242	TCAATATCAAACCTGATTTT	
57	-	68710	68729	9.45e-05	0.244	ATAAATGCTTATCTGTTTTC	

[a]The motif is ranked by increasing p-value.

[b]The probability of a random sequence of the same length as the motif matching that position of the sequence with as good/better score.

[c]The false discovery rate if the occurrence is accepted as significant.

Supplementary Table S5. Oligonucleotides used in this study. A list of the oligonucleotides and some additional information (e.g., sequence, the purpose they were used for, reference) are provided.

Oligo	Sequence (5' -3')	Target	Method	Description	Reference
5R_L	GUGAUCCAACCGACGCGACAAGCUAAUGCAAGANNN		5' RACE	5'-RACE RNA linker	Zhelyazkova et al., 2013
5R_1	TGATCCAACCGACGCGAC	5'-RACE RNA linker		nested PCR1 linker specific primer	Zhelyazkova et al., 2013
5R_2	GATCCAACCGACGCGACA			nested PCR2 linker specific primer	Zhelyazkova et al., 2013
PB1	GTCCCGGCAAGGTAAAAAC	aTSS_925, aTSS_9633, aTSS_9764, aTSS_39561, aTSS_39430		cDNA synthesis & nested PCR1 target specific primer	This study
PB2	GGCAAGGTAAAAACCTTGAAGC	aTSS_925, aTSS_9764, aTSS_39430		nested PCR2 target specific primer	This study
PB3	AGGGGGTAACAACCCCTTTG	gTSS_1994, gTSS_1987		cDNA synthesis & nested PCR1 target specific primer	This study
PB4	CTGCGTTCCAACCGCTACC			nested PCR2 target specific primer	This study
PB5	TGTTACCCCCTCCACACCT	aTSS_2433		cDNA synthesis & nested PCR1 target specific primer	This study
PB6	CCCTCCACACCTGTAAATACGA			nested PCR2 target specific primer	This study
PB7	GCAGTTCGGCTTCGTGAAAG	aTSS_9633, aTSS_39561		nested PCR2 target specific primer	This study
PB8	CTCCTGAGTCTCTCGCACAA	gTSS_10680, iTSS_10680		cDNA synthesis & nested PCR1 target specific primer	This study
PB9	AAATCCATATCTGAGGCTATTTTGA			nested PCR2 target specific primer	This study
PB10	AATCCCCGTTTCACAATCAT	iTSS_12275		cDNA synthesis & nested PCR1 target specific primer	This study
PB11	GGAGCACAGCTTTTTCCACA			nested PCR2 target specific primer	This study
PB12	AAAATAACGACGTTTATGCATGT	oTSS_13268		cDNA synthesis & nested PCR1 target specific primer	This study
PB13	CTGGCCTGCTGCCGTAGATA			nested PCR2 target specific primer	This study
PB14	TACACCGCAGGACATCGGTA	iTSS_19566, iTSS_20268, iPS_19439		cDNA synthesis & nested PCR1 target specific primer	This study
PB15	TCCTGATAAGGCGATACATGTCC	iTSS_19566		nested PCR2 target specific primer	This study
PB16	CGCAACAGATCCCCACTCAC	iTSS_20268		nested PCR2 target specific primer	This study
PB17	GTGAGCTCATTCCAGGCTGA	aTSS_26234		cDNA synthesis & nested PCR1 target specific primer	This study
PB18	CTGACAATATCATTGTGCACCGTA			nested PCR2 target specific primer	This study
PB19	CATGACCTTCGACAACACCA	gTSS_17351		cDNA synthesis & nested PCR1 target specific primer	This study
PB20	GCCGAAATTCTGCTGAACC			nested PCR2 target specific primer	This study
PB21	TTGATCAGATCGCACAATCC	oTSS_74127		cDNA synthesis & nested PCR1 target specific primer	This study
PB22	GGGCTGAGTTCCCTCAAAGTG			nested PCR2 target specific primer	This study
PB23	TCATAGTTGCTTCATCTGATTTTT	iTSS_22022		cDNA synthesis & nested PCR1 target specific primer	This study
PB24	TTTTCCGCTAACTTCTTCTTTAGCC			nested PCR2 target specific primer	This study
PB25	CTTCGTTCTGCATGGTCGTA	gTSS_30158		cDNA synthesis & nested PCR1 target specific primer	This study
PB26	CAAAAATCAGCGCGTTAATACACA			nested PCR2 target specific primer	This study
PB27	ACCAGGGTTTCAGGGTGTTC	iTSS_44859, gTSS_44859, gPS_44802		cDNA synthesis & nested PCR1 target specific primer	This study
PB28	CGAATCATCCTGTTACGGTCA	iTSS_44859, gTSS_44859		nested PCR2 target specific primer	This study
PB29	GCTGACATGTTTGGCGGTAA	gTSS_55560, iPS_55691		cDNA synthesis & nested PCR1 target specific primer	This study
PB30	CATAGGATGTGGATGGAGCAGTC			nested PCR2 target specific primer	This study
PB31	GCTGCGAGATAATAGGCAAAA	gTSS_66404		cDNA synthesis & nested PCR1 target specific primer	This study
PB32	ACCATAACGACTGTTCCTCCTTG			nested PCR2 target specific primer	This study

Oligo	Sequence (5' -3')	Target	Method	Description	Reference
PB33	TCATGCAAGCTTTCTCTTGAA	gTSS_67507	5' RACE	cDNA synthesis & nested PCR1 target specific primer	This study
PB34	CCATATTTCTTTTCATGGCCACA			nested PCR2 target specific primer	This study
PB35	CGATCTGATCAATCACCCAAA	oTSS_73951, oTSS_73955		cDNA synthesis & nested PCR1 target specific primer	This study
PB36	TCTGATCAATCACCCAAAAGAAAA			nested PCR2 target specific primer	This study
PB37	AAAAAGCCTCCATCAGAGAGGA	aTSS_44129		cDNA synthesis & nested PCR1 target specific primer	This study
PB38	TCAGAGAGGAGGCAGGGAAA			nested PCR2 target specific primer	This study
PB39	CATTGCTCTATTCATGCACCA	gPS_195		cDNA synthesis & nested PCR1 target specific primer	This study
PB40	GGCCAGTTTCCAGAGCTCAA			nested PCR2 target specific primer	This study
PB41	CTGAAACACACGGTGGCAGT	gPS_7674		cDNA synthesis & nested PCR1 target specific primer	This study
PB42	TGAGATAACCTCAAAAGCCCGTA			nested PCR2 target specific primer	This study
PB43	CAACCGTACCAAATGGCAAAA	iPS_19439		nested PCR2 target specific primer	This study
PB44	TTTCTCCAGAAGAATTCGGCTTA	gPS_22460		cDNA synthesis & nested PCR1 target specific primer	This study
PB45	TGGGCATGCGCAAAAGTAAA			nested PCR2 target specific primer	This study
PB46	GTTCTCATCGGCAAAAGAGC	gPS_29961		cDNA synthesis & nested PCR1 target specific primer	This study
PB47	ACGTCATACGCCTTGAGCAG			nested PCR2 target specific primer	This study
PB48	AAGCTCTTTTACGGGCCACT	gPS_38370		cDNA synthesis & nested PCR1 target specific primer	This study
PB49	CGGATTCCCCTTTCTGTATGC			nested PCR2 target specific primer	This study
PB50	CGTCTCCCCTTCCGTTTTTC	gPS_44802		nested PCR2 target specific primer	This study
PB51	CTGGCCATGCCGAAGAAAGT	iPS_55691		nested PCR2 target specific primer	This study
PB52	CAGGCTCACGGAGTTCTTTC	iPS_59728		cDNA synthesis & nested PCR1 target specific primer	This study
PB53	CACGGAGTTCTTTCAGAAAAAGG			nested PCR2 target specific primer	This study
PB54	TCAGGATTCTCTGATTAATGTAAAGC	aPS_66465		cDNA synthesis & nested PCR1 target specific primer	This study
PB55	GGATTCTCTGATTAATGTAAAGCAAAA			nested PCR2 target specific primer	This study
PB56	caggaatagctGGAGCGTCA	aPS_19452		cDNA synthesis & nested PCR1 target specific primer	This study
PB57	TGGCTGGCCTGGAGTCAGTA			nested PCR2 target specific primer	This study
3R_1	TTCCTGTTACCGTTCGACTTG	*rrn16*	3' RACE	cDNA synthesis & nested PCR1 *rrn16* (linker) specific primer	This study
3R_2	TTACCGTTCGACTTGCATGTGT			nested PCR2 *rrn16* (linker) specific primer	This study
PB58	TGGACTCCAACCTTATTTGTCTT	aTSS_2433		nested PCR1 target specific primer	This study
PB59	TTTACAGGTGTGGAGGGGGTA			nested PCR2 target specific primer	This study
PB60	GTGTCTGGGCGATACTCAGG	oTSS_6357		nested PCR1 target specific primer	This study
PB61	ACCGTTCGTGAATGCAAAGC			nested PCR2 target specific primer	This study
PB62	TCCATGCTGCAGTTGATTTT	gTSS_22460		nested PCR1 target specific primer	This study
PB63	GCATCCTGCCAGTCACTCTG			nested PCR2 target specific primer	This study
PB64	GCTGTGCCAGCACCTCTG	aTSS_45870		nested PCR1 target specific primer	This study
PB65	AGCACCTCTGCCGTGACC			nested PCR2 target specific primer	This study
PB66	GCCAGACACTGCTGTTGTTC	aTSS_57462		nested PCR1 target specific primer	This study
PB67	ATCGGAGAAAAACGGGGAAT			nested PCR2 target specific primer	This study
PB68	TCATGCTCAGAGCCATTCC	aTSS_59447		nested PCR1 target specific primer	This study
PB69	CGGTCTTCCCAGCTAAAAGC			nested PCR2 target specific primer	This study
PB70	CCTGGACGGTCACTTCTGAG	oTSS_59885		nested PCR1 target specific primer	This study
PB71	AACAGGCTCACGGAGTTCTTTC			nested PCR2 target specific primer	This study
PB72	CTGGGGATTGGTGCTATGAT	oTSS_73951		nested PCR1 target specific primer	This study
PB73	CGCTCAGTCCGCTTGGTAAT			nested PCR2 target specific primer	This study

Oligo	Sequence (5' -3')	Target	Method	Description	Reference
PB74	CGGGTTAAAAAGCAGCCTTA	*sepA*	Cloning	forward primer for cloing of *sepA* under native promoter	This study
PB75	GCTCTAGACAGGCTCACGGAGTTCTTTC			reverse primer for cloning of *sepA* under native promoter, red = XbaI restriction site	This study
PB76	CATCTTGCGTTTTTTTTGGTTG			forward primer for cloing of *sepA* under native promoter (excluding the upstream predicted AggR binding site)	This study
PB77	CTGGCAGTTCCCTACTCTCG			reverse primer for cloing of *sepA* under native promoter (excluding the upstream predicted AggR binding site)	This study
PB76	GGGGTACCCCCGCAGAGTTGCCTGATAAAG	*aggR*		forward primer for cloning of *aggR* under inducible promoter, red = KpnI restriction site	This study
PB77	GCTCTAGAGCACGCTGGACATGAGATAACC			reverse primer for cloning of *aggR* under inducible promoter, red = XbaI restriction site	This study
GapA_for	GTTGTCGCTGAAGCAACTGG	*gapA*	RT-PCR	forward primer	Blumer et al., 2005
GapA_rev	AGCGTTGGAAACGATGTCCT			reverse primer	Blumer et al., 2005

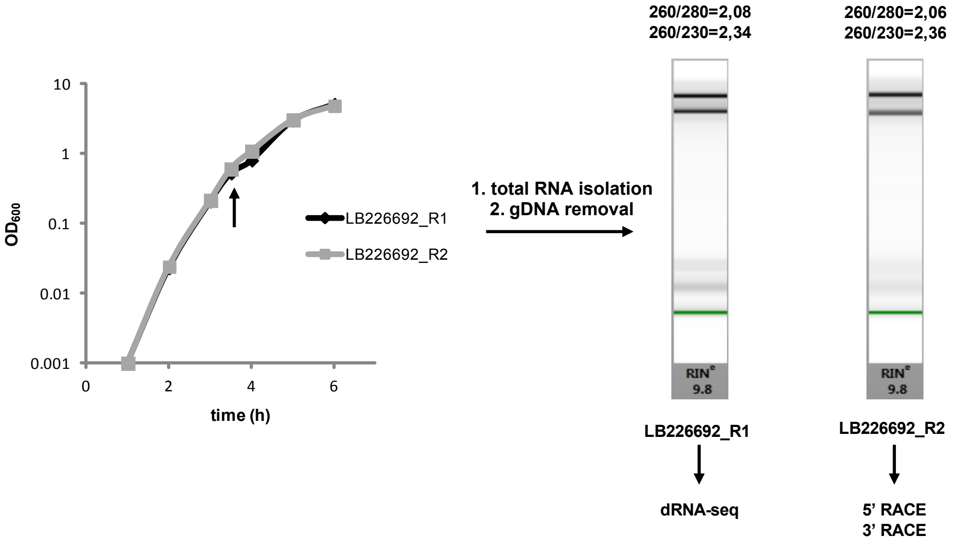

Supplementary Figure S1. RNA sample preparation for transcriptome analysis of the *E. coli* O104:H4 pAA plasmid. Exponentially growing cells of the *E. coli* O104:H4 clinical isolate LB226692 were harvested at OD600 of 0.52 (LB226692_R1, replicate 1) and 0.6 (LB226692_R2, replicate 2; black arrow in the growth curve). Total RNA was isolated and the gDNA was removed. Concentration and purity of the RNA samples was determined using Nano Drop. The 260/280 and 260/230 ratios are shown. The RNA integrity was monitored on the Agilent 2200 TapeStation. Major RNA bands and the RNA integrity number (RIN) are shown. One of the biological replicates was used for dRNA-seq and the other one for 5' RACE and 3' RACE analysis.

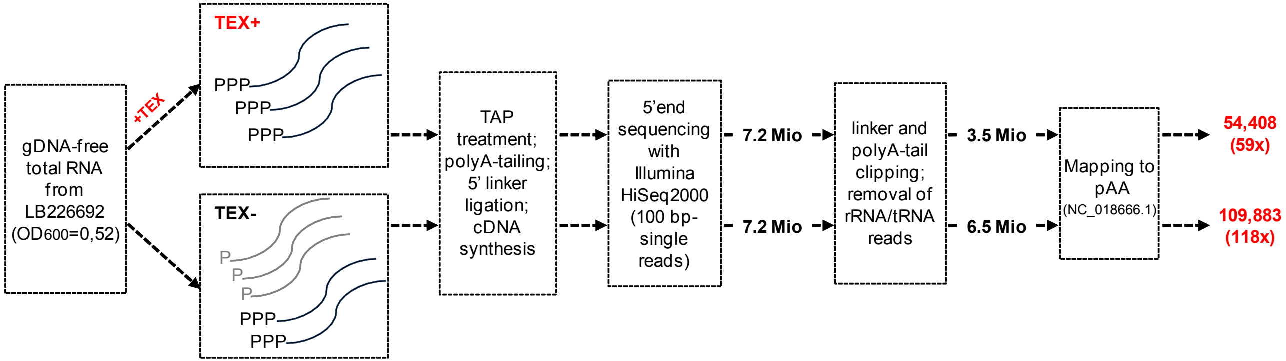

Supplementary Figure S2. dRNA-seq experimental set up and an overiview of sequenced and mapped reads. Equal amounts of gDNA-free total RNA isolated from exponentially growing cells of the *E. coli* O104:H4 strain LB226692 were incubated with TEX+ (5'-P depleted RNA) or left untreated (containing both 5'-PPP and 5'-P RNAs). Next, RNA was treated with tobacco acid pyrophosphatase (TAP), which converts 5'-PPP to 5'-P, poly(A)-tailed using poly(A) polymerase and a 5' end RNA adapter was ligatated. First-strand cDNA was synthesized using an oligo(dT)-adapter primer and the M-MLV reverse transcriptase. Sequencing was performed on a HiSeq 2500 machine (Illumina) in a 100 bp single-end read mode. Approximately 7.2 million reads were obtained from each library. After linker and poly A-tail removal rRNA and tRNA reads were filtered out. The resulting 3.5 and 6.5 million reads for the TEX+ and TEX- library, respectively, were mapped to the *E. coli* O104:H4 genome consisting of the chromosome and the plasmids pESBL, pG and pAA. In total, 54,408 and 109,883 reads were mapped onto pAA (NC_018666.1) in TEX+ and TEX- library, which correspond to coverage of 59x and 118x, respectively.

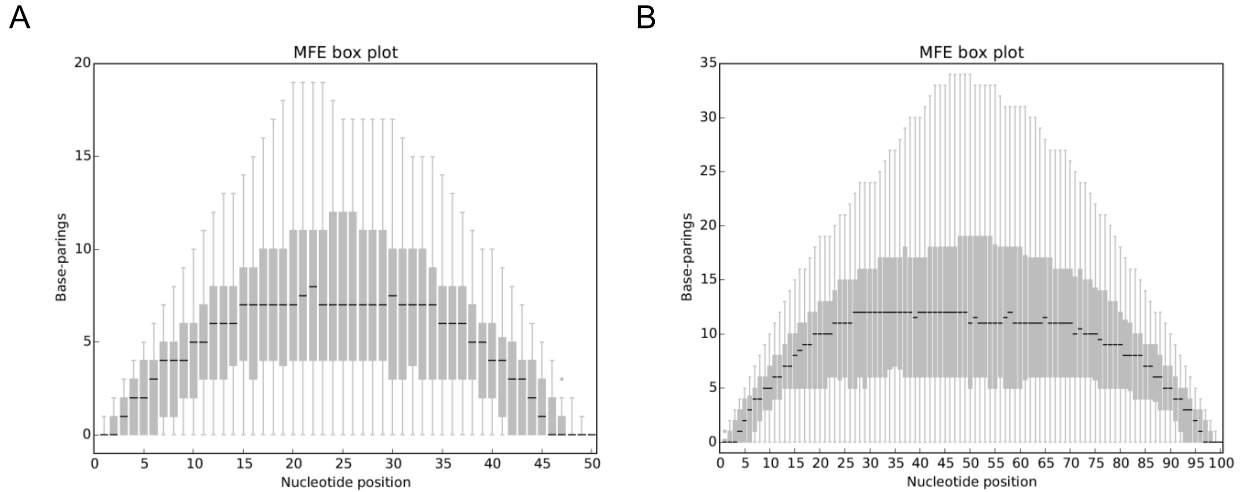

Supplementary Figure S3. RNA secundary structure prediction of the downstream regions of TSS. The 50 **(A)** and 100 **(B)** nucleotide-long regions downstream of the pAA-associated TSS mapped by dRNA-seq were subjected to RNA secondary structure prediction using RNAfold. Mountain plot values were calculated based on the minimum free energy structures predicted for the analyzed sequences and diagrams representing the number of enclosing nucleotides per nucleotide position were generated with Python. Boxes represent the lower to upper quartile values, the line stands for the median and the whiskers for the upper and lower value of the range of the data. The analysis revealed a low probability for the occurrence of base pairings immediately downstream of the mapped TSS.

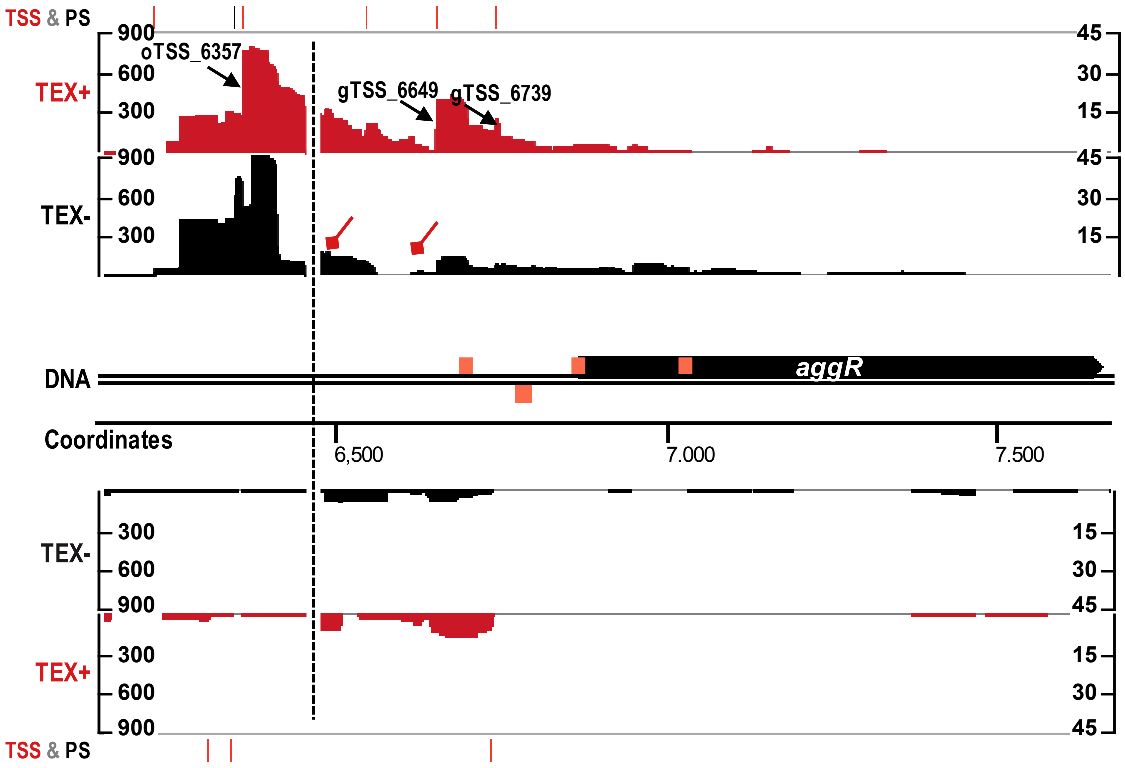

Supplementary Figure S4. The transcriptome profile of *aggR*. cDNA reads from TEX+ (red) and TEX- (black) libraries mapped against *aggR* are shown. Due to differential abundance of associated cDNA reads the dRNA-seq graphs are split in two parts (dashed vertical line) and the y-axis (abundance relative score) for each part is given. Annotated TSS (red) and PS (black) are shown above the dRNA-seq graphs. TSS candidates discussed in the main text are indicated in the dRNA-seq graphs with black arrows. The approx. position of 3' ends determined by 3' RACE are indicated by red diamond arrows. Predicted AggR binding sites are represented on the DNA strand as orange boxes.

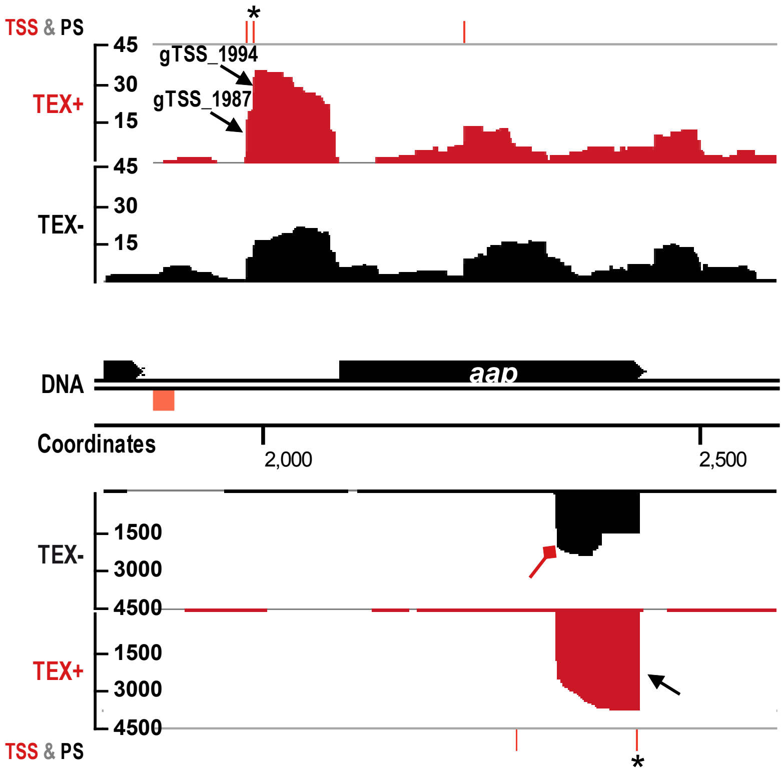

Supplementary Figure S5. The transcriptome profile of *aap*. cDNA reads from TEX+ (red) and TEX- (black) libraries mapped against the *aap* gene coding for dispersin are shown. Annotated TSS (red) and PS (black) are shown above the dRNA-seq graphs and the ones subjected to 5' RACE verification in a biological replicate are marked with an asterisk. TSS candidates discussed in the main text are indicated in the dRNA-seq graphs with black arrows. The approx. position of 3' ends determined by 3' RACE are indicated by red diamond arrows. The predicted AggR binding site is represented on the DNA strand as an orange box.

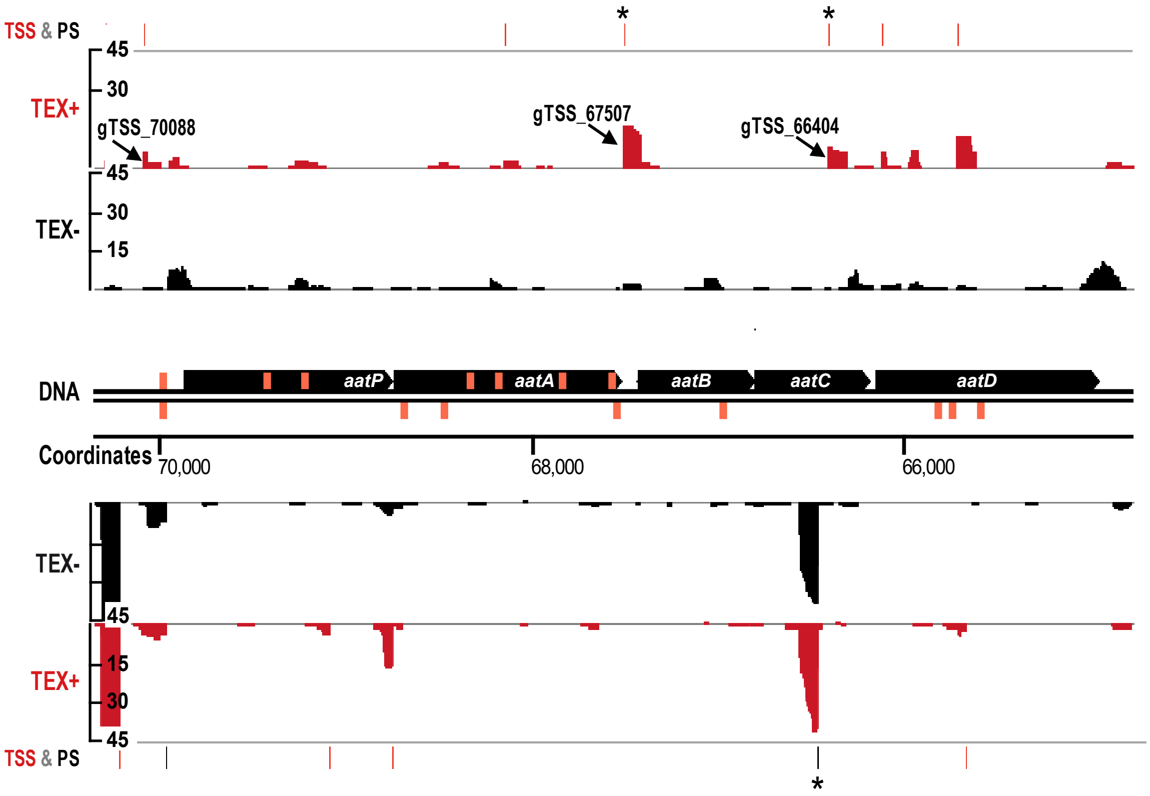

Supplementary Figure S6. The transcriptome profile of *aatPABCD*. cDNA reads from TEX+ (red) and TEX- (black) libraries mapped against the *aat* gene cluster coding for the dispersin secretion system are shown. Annotated TSS (red) and PS (black) are shown above the dRNA-seq graphs and the ones subjected to 5' RACE verification in a biological replicate are marked with an asterisk. TSS candidates discussed in the main text are indicated in the dRNA-seq graphs with black arrows. The approx. position of 3' ends determined by 3' RACE are indicated by red diamond arrows. Predicted AggR binding sites are represented on the DNA strand as orange boxes.

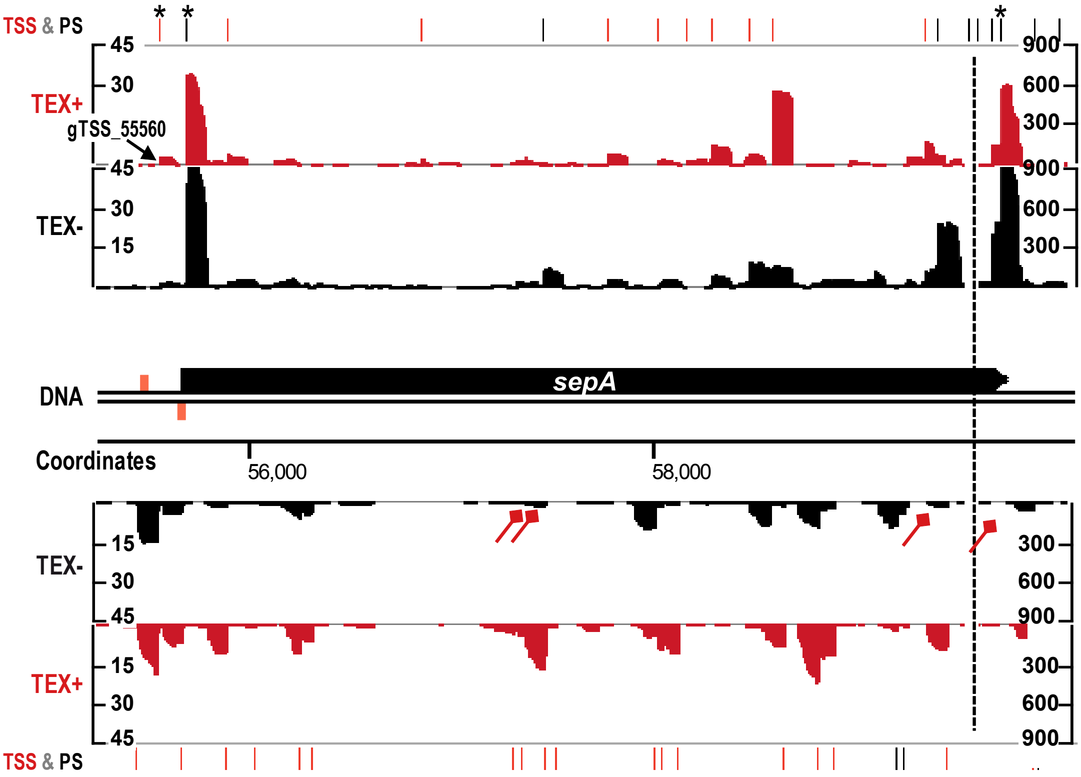

Supplementary Figure S7. The transcriptome profile of *sepA*. cDNA reads from TEX+ (red) and TEX- (black) libraries mapped against the *sepA* gene coding for the serine protease SepA are shown. Due to differential abundance of associated cDNA reads the dRNA-seq graphs in (A) and (D) are split in two parts (dashed vertical line) and the y-axis (abundance relative score) for each part is given. Annotated TSS (red) and PS (black) are shown above the dRNA-seq graphs and the ones subjected to 5' RACE verification in a biological replicate are marked with an asterisk. TSS candidates discussed in the main text are indicated in the dRNA-seq graphs with black arrows. The approx. position of 3' ends determined by 3' RACE are indicated by red diamond arrows. Predicted AggR binding sites are represented on the DNA strand as orange boxes.

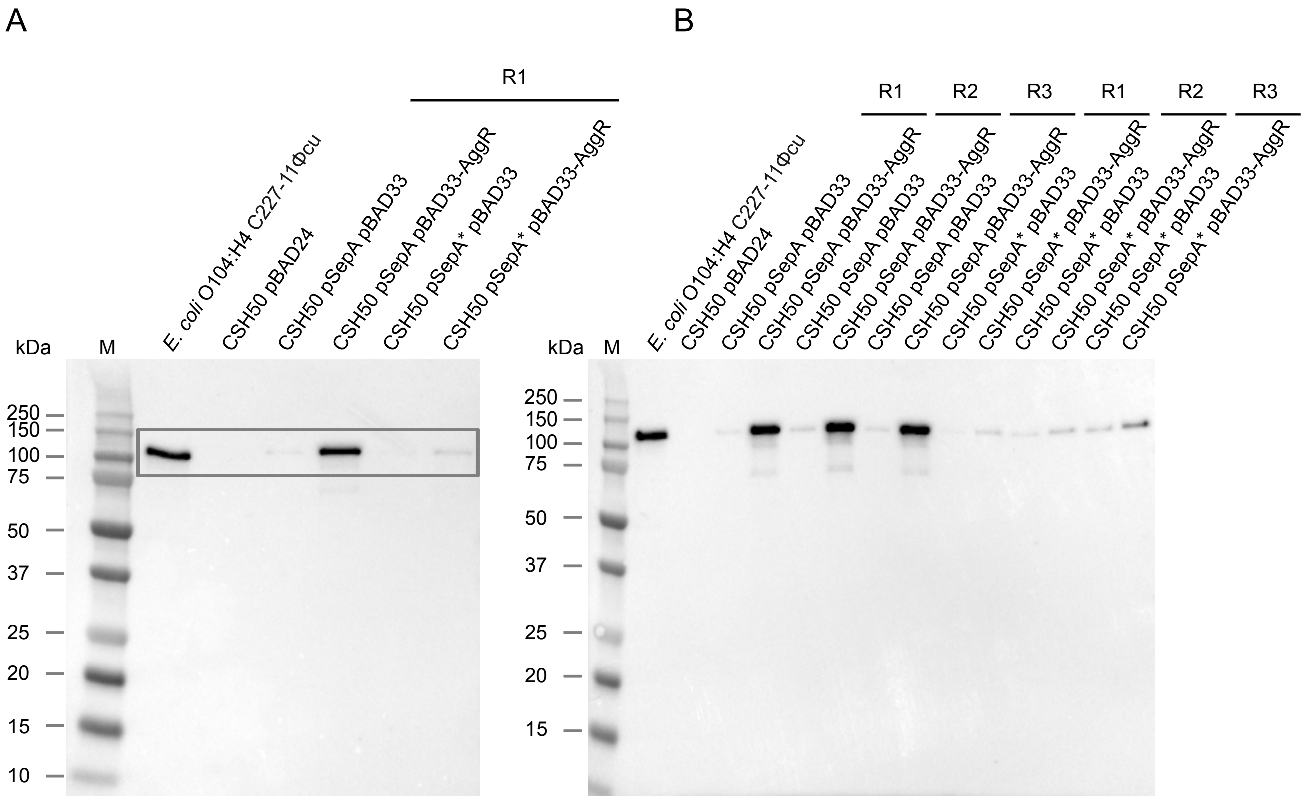

Supplementary Figure S8. AggR-dependent *sepA* activation. A. Full-length versions of the immunoblot presented in Figure 5A. The cropped area of interest in Figure 5 is indicated by a boxe. **B. The gel of the three biological replicates used for the quantification of the AggR-dependent *sepA* activation.** The blot with the three biological replicates (R1-R3) used for the quantification of the AggR-dependent *sepA* activation (Figure 5B) is shown. The sizes of the bands of the protein marker (Precision Plus Protein Dual Color, Bio-Rad) are given.
